# Ndrg1 is a T-cell clonal anergy factor negatively regulated by CD28 costimulation and interleukin-2

**DOI:** 10.1038/ncomms9698

**Published:** 2015-10-28

**Authors:** Yu Mi Oh, Hyung Bae Park, Jae Hun Shin, Ji Eun Lee, Ha Young Park, Dhong Hyo Kho, Jun Sung Lee, Heonsik Choi, Tomohiko Okuda, Koichi Kokame, Toshiyuki Miyata, In-Hoo Kim, Seung Hoon Lee, Ronald H. Schwartz, Kyungho Choi

**Affiliations:** 1 Department of Biochemistry and Molecular Biology, Seoul National University College of Medicine, 103 Daehak-ro, Chongno-gu, Seoul 110-799, Korea; 2 Department of Biomedical Sciences, Seoul National University College of Medicine, 103 Daehak-ro, Chongno-gu, Seoul 110-799, Korea; 3 Specific Organs Cancer Branch, Research Institute National Cancer Center, Gyeonggi-do 410-769, Korea; 4 Hematologic Malignancy Branch, Research Institute National Cancer Center, Gyeonggi-do 410-769, Korea; 5 Laboratory of Cellular and Molecular Immunology, National Institute of Allergy and Infectious Diseases, National Institutes of Health, Bethesda, Maryland 20892, USA; 6 Department of Molecular Pathogenesis, National Cerebral and Cardiovascular Center, Suita, Osaka 565-8565, Japan; 7 Molecular Imaging and Therapy Branch, Research Institute National Cancer Center, Gyeonggi-do 410-769, Korea

## Abstract

Induction of T-cell clonal anergy involves serial activation of transcription factors, including NFAT and Egr2/3. However, downstream effector mechanisms of these transcription factors are not fully understood yet. Here we identify Ndrg1 as an anergy factor induced by Egr2. Ndrg1 is upregulated by anergic signalling and maintained at high levels in resting anergic T cells. Overexpression of Ndrg1 mimics the anergic state and knockout of the gene prevents anergy induction. Interestingly, Ndrg1 is phosphorylated and degraded by CD28 signalling in a proteasome-dependent manner, explaining the costimulation dependence of anergy prevention. Similarly, IL-2 treatment of anergic T cells, under conditions that lead to the reversal of anergy, also induces Ndrg1 phosphorylation and degradation. Finally, older Ndrg1-deficient mice show T-cell hyperresponsiveness and Ndrg1-deficient T cells aggravate inducible autoimmune inflammation. Thus, Ndrg1 contributes to the maintenance of clonal anergy and inhibition of T-cell-mediated inflammation.

## Abstract (web summary)

T cell clonal anergy is mediated by Egr2 but its downstream effectors have not been characterized. Here the authors show that Egr2 activates Ndrg1, which is critical for the anergy state, while anergy-counteracting signals IL-2 or CD-28 promote Ndrg1 phosphorylation and degradation.

## Graphical abstract

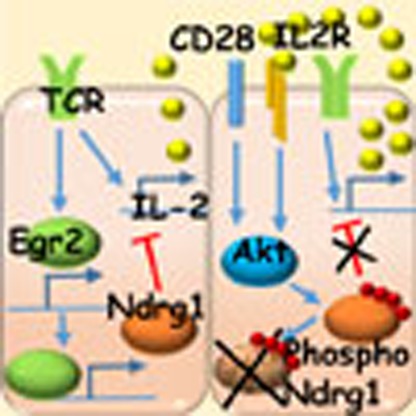

T cell clonal anergy is defined as a hyporesponsive state of T cells following T-cell receptor (TCR) engagement in the absence of costimulation^1^. Anergic T cells proliferate poorly and produce little interleukin (IL)-2 on subsequent TCR stimulation, even in the presence of costimulation. It has long been thought that T-cell clonal anergy might represent a peripheral tolerance mechanism in which autoreactive naive T cells escaping the thymus could be rendered unresponsive following recognition of self-antigens on antigen-presenting cell (APC) in the absence of inflammation^2^. However, T-cell clonal anergy is primarily an *in vitro* phenomenon of ‘T-cell clones', which have extensively experienced the antigen, and not of antigen-inexperienced naive T cells^3^. Thus, the presumption that anergy of T-cell clones is a model of naive T-cell tolerance was questioned, which has raised some doubt as to whether clonal anergy has any physiologic relevance for T-cell tolerance *in vivo*^4^. Therefore, identification of molecular mechanisms specific for T-cell clonal anergy and manipulation of such molecules *in vivo* could have important value for understanding the role of ‘clonal' anergy in T-cell tolerance.

For the induction of T-cell clonal anergy, the importance of the calcium–calcineurin–NFAT signalling pathway is clear. Treatment with the calcineurin inhibitor cyclosporine A prevented anergy induction^5^ and calcium influx using ionomycin induces an anergy-like state^6^,^7^. Furthermore, *NFAT*^−/−^ T cells are resistant to anergy induction^7^. It is also known that treatment during the induction phase with cycloheximide, a protein synthesis inhibitor, prevents anergy, suggesting that the synthesis of new protein(s) (also called anergy factor(s)) is required for anergy induction^8^. As NFAT is a transcription factor, the anergy factors are believed to be induced by NFAT.

Egr2/3 is one such protein family that is induced by NFAT^9^ and found to be upregulated under anergy-inducing conditions^10^. Egr3 knockout T cells are resistant to anergy induction *in vivo* and small interfering RNA-mediated knockdown of Egr2 in a T-cell clone inhibited full induction of anergy^10^,^11^. However, the fact that Egr2 and 3 are also transcription factors raises the possibility that further downstream effector molecules, executing inhibitory action on TCR signalling during antigen rechallenge, could be induced by Egr2/3. Cbl-b and DGK-α, in this sense, were proposed as targets of Egr2/3 (refs 10, 12). They were induced by TCR stimulation alone or by ionomycin treatment^7^,^10^,^13^. Knockout T cells for these molecules were resistant to *in vitro* anergy and various models of *in vivo* anergy^14^,^15^. However, these knockout T cells showed increased reactivity of naive T cells *per se* without the induction of anergy^15^,^16^, whereas Egr2 or Egr3 knockout T cells only showed enhanced responsiveness after anergy induction^10^,^17^,^18^. Therefore, Cbl-b and DGK-α may be involved not only in the anergic phenotype, but also in a general negative regulation of T-cell activation. Thus, anergy-specific effector molecules downstream of Egr2/3 need to be further identified.

T-cell clonal anergy is conceptually based on the two-signal model of cell activation^19^. According to this model, signal 1 (TCR signal) plus signal 2 (costimulation) generates productive activation of T cells, whereas signal 1 in the absence of signal 2 leads to the induction of anergy. In other words, signal 2 is necessary for prevention of anergy. Thus, in molecular terms, any anergy factors induced by signal 1 should be inactivated by signal 2 (ref. 19). The most well-studied signal 2-inducing molecule on the T-cell surface is CD28. Addition of agonistic antibody to CD28 during the induction phase prevents anergy^20^ and supplying CTLA4-Ig, which blocks CD28 signals during a productive stimulation, results in anergy induction^21^. The molecular mechanism underlying this phenomenon, however, has not been clearly defined.

In the present work, we pursued new effector molecules of T-cell clonal anergy using two independent microarray approaches. We identified one gene, named *Ndrg1*, that was upregulated under anergy-inducing conditions and showed delayed induction kinetics compared with Egr2. It remained upregulated in resting anergic CD4^+^ T cells. In other cell types, Ndrg1 is a cytosolic protein that has been reported to be upregulated by various stress signals, such as hypoxia, endoplasmic reticulum (ER) stress and DNA-damaging reagents^22^,^23^. Here we provide experimental evidence that Ndrg1 is an anergy factor in CD4^+^ T cells, which is induced by Egr2 and inhibited by CD28-mediated costimulation.

## Results

### Ndrg1 is upregulated under anergy-inducing conditions

**Figure 1:**
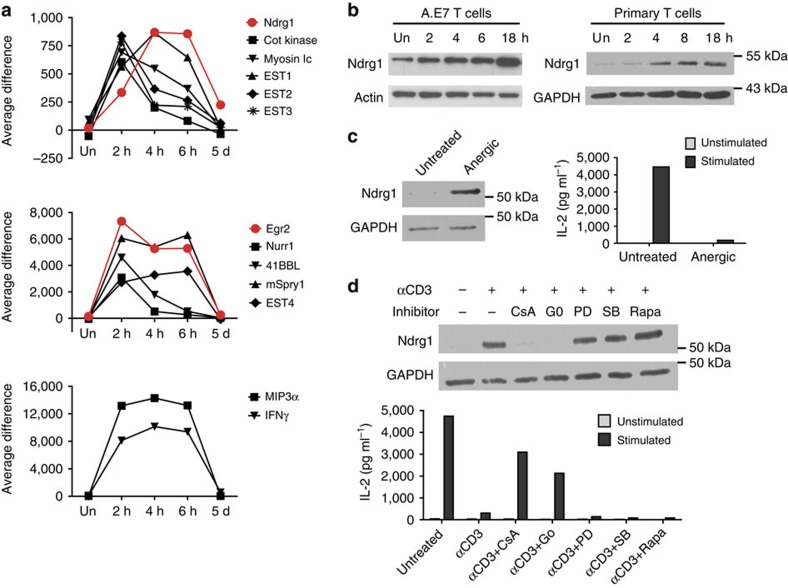
Ndrg1 is upregulated by an anergic stimulus and maintained in the resting anergic state. (**a**) Kinetic analysis of gene expression following an anergic stimulus using oligonucleotide microarray chips. Biotinylated RNA probes prepared from A.E7 cells stimulated with anti-TCR for 2, 4 and 6 h or stimulated for 18 h followed by 5 days resting, were hybridized to Affymetrix gene chips. The amounts of mRNA are represented as the fluorescence intensity (average difference of 16 signals per gene). (**b**) Immunoblot analysis of Ndrg1 in A.E7 cells and pre-activated CD4^+^ primary cells (from 5CC7^+^ TCR transgenic mice) stimulated with anti-TCR for various time points. Un, unstimulated. (**c**) Resting A.E7 cells (untreated) or A.E7 cells made anergic by anti-TCR treatment for 18 h and resting for 5 days (anergic) were analysed by immunoblotting of Ndrg1(left panel) or stimulated with anti-CD3 and anti-CD28 for 48 h, to measure IL-2 production (right panel). (**d**) A.E7 cells treated with anti-CD3 plus various reagents for 18 h were subject to immunoblot analysis of Ndrg1 (upper blot) or washed and rested for 5 days before being stimulated with anti-CD3 and CD28, to measure IL-2 production (lower graph). CsA, cyclosporine A; Go, Gö6976; PD, PD98059; SB, SB203580; Rapa, rapamycin. Actin or glyceraldehydes 3-phosphate dehydrogenase (GAPDH) was used as a loading control for the immunoblotting. Results are representative of one (**a**) or two independent experiments (**b**–**d**).

A previous DNA microarray screening to find anergy factors identified Egr2/3 as the major transcription factors governing anergy programmes^10^,^24^. We attempted to pull out candidate effector molecules from the same DNA microarray sets used to identify Egr2/3. In this screening, A.E7, a CD4^+^ T-cell clone, was treated with agonistic anti-TCR antibody, to induce anergy either with anergy-blocking reagents (cyclosporin A and Gö6976) or with non-blocking chemicals (ref. 10 and Fig. 1d). Messenger RNAs that were upregulated by anti-TCR alone or with irrelevant chemicals but were not induced by anti-TCR in the presence of anergy-blocking reagents were considered as potential anergy factors. Approximately 1,260 genes showed more than 3-fold upregulation of mRNA by anti-TCR alone. We applied highly stringent criteria using the above conditions to narrow down candidate genes and ended up with 13 genes (Supplementary Table 1). It was known that anergy induction requires at least 4–6 h of anti-TCR treatment to complete^5^. Therefore, we performed another set of DNA microarrays, to examine the kinetics of upregulation^25^. We isolated mRNAs from A.E7 cells treated with anti-TCR for 2, 4 or 6 h. In addition, mRNAs were prepared from anergic A.E7 cells that were treated with anti-TCR for 18 h followed by 5 days of resting in the absence of stimulation. Out of the 13 candidate genes, 11 mRNAs, including Egr2, showed peak induction at 2 h and were either maintained or declined after 2 h. After 5 days of rest, these were all completely downregulated (Fig. 1a). In contrast, two mRNAs were more slowly upregulated and reached a peak level at 4 h, showing delayed induction compared with Egr2. However, only one mRNA, Ndrg1, was maintained at a relatively high level in the resting anergic state. This delayed but persistent expression of Ndrg1 mRNA fits well with the process of anergy induction. Protein induction was delayed even more than mRNA induction, reaching a peak level at 18 h in A.E7 cells and in pre-activated primary CD4^+^ T cells as well (Fig. 1b). Most importantly, similar to the level of mRNA, Ndrg1 protein was upregulated in resting anergic A.E7 cells, at which time the cells showed a profound defect in IL-2 secretion following TCR stimulation (Fig. 1c). Furthermore, Ndrg1 protein induction was inhibited by anergy blockers, but not inhibited by non-blocking chemicals (Fig. 1d). Thus, we conclude that Ndrg1 is upregulated by an anergic stimulus and maintained in resting anergic T cells.

### Ndrg1 overexpression mimics the anergic state

**Figure 2:**
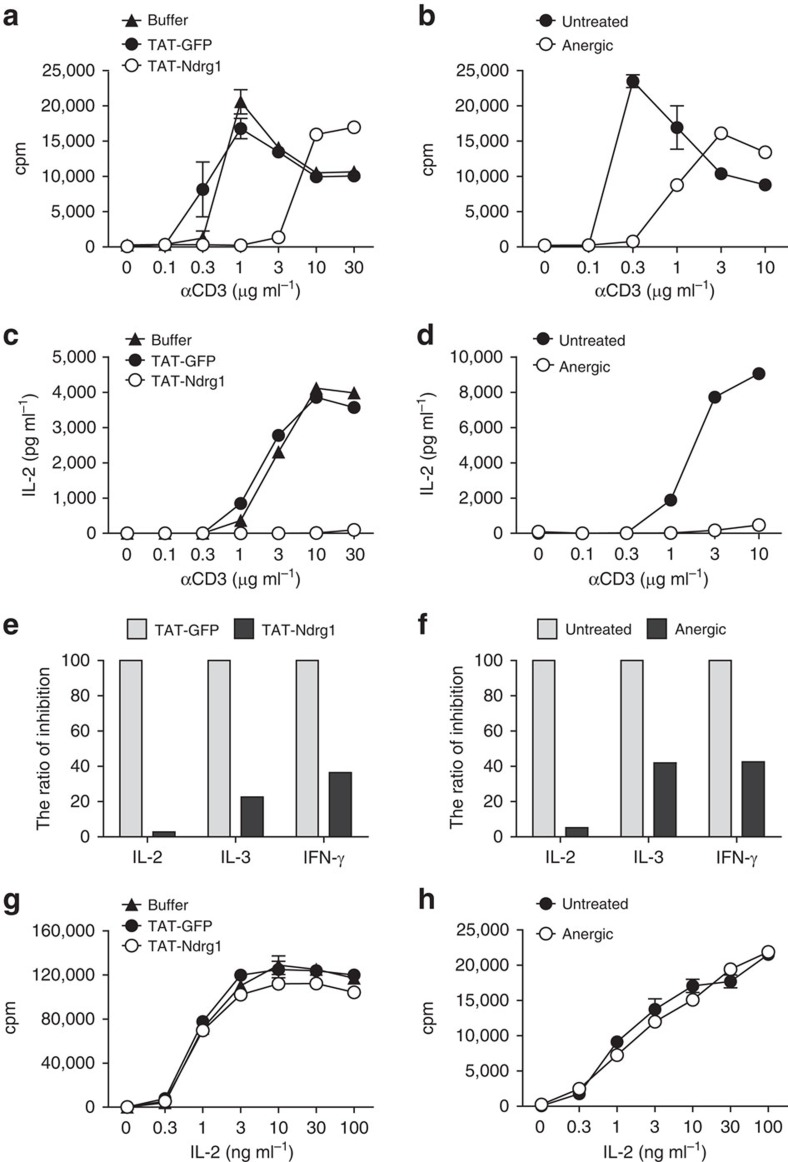
Overexpression of Ndrg1 in a T-cell clone mimics the anergic state. (**a**,**b**) Proliferation of TAT-protein-transduced A.E7 cells (**a**) or anergic A.E7 cells (**b**) stimulated with various concentrations of anti-CD3 plus anti-CD28 for 48 h was measured in a ^3^H-thymidine incorporation assay. Anergic A.E7 cells were generated by 18 h stimulation with anti-CD3 and 5 days resting. (**c**,**d**) IL-2 production of TAT-protein-transduced A.E7 cells (**c**) or anergic A.E7 cells (**d**) stimulated as in **a**,**b** was measured by ELISA assay. (**e**,**f**) The relative inhibition of cytokine production in TAT–Ndrg1-transduced A.E7 cells (**e**) or anergic A.E7 cells (**f**) stimulated with 30 (**e**) or 10 μg ml^−1^ (**f**) of anti-CD3 plus anti-CD28 for 48 h. The ratio of inhibition=(amount of cytokine produced by TAT–Ndrg1-transduced cells or anergic cells/amount of cytokine produced by TAT–GFP-transduced cells or untreated cells) × 100. (**g**,**h**) IL-2-induced proliferation of TAT–Ndrg1-transduced A.E7 cells (**g**) or anergic A.E7 cells (**h**) was measured in a ^3^H-thymidine incorporation assay after 48 h treatment with various concentrations of IL-2. Results are representative of two independent experiments (**a**–**h**); error bars, s.d.

To determine whether Ndrg1 plays a role in T-cell anergy, we first carried out a gain-of-function study. Ndrg1 was overexpressed in A.E7 T cells using TAT-fusion protein transduction. This technique has been successfully used previously for overexpression of proteins in T-cell clones and primary T cells^25^. TAT–Ndrg1-transduced A.E7 cells were stimulated with anti-CD3 plus anti-CD28, the stimulus used for restimulation of anergic T cells. The proliferation of TAT–Ndrg1-overexpressing cells was significantly attenuated compared with that of control cells expressing green fluorescent protein (GFP) (Fig. 2a). IL-2 production was more profoundly affected by Ndrg1 overexpression showing almost complete inhibition (Fig. 2c). This pattern of proliferation and IL-2 production was comparable to that seen for A.E7 cells anergized by anti-CD3 (Fig. 2b,d). The inhibitory effect of TAT–Ndrg1 was also observed in primary naive T cells and pre-activated T cells (Supplementary Fig. 1). For anergic A.E7 cells, interferon (IFN)-γ and IL-3 production are less affected than IL-2 production (Fig. 2f). Ndrg1-overexpressing cells showed a similar degree of reduction in IFN-γ and IL-3 production, mimicking the cytokine hierarchy seen for anergic cells (Fig. 2e). Finally, even though anergic T cells have reduced proliferative capacity, they can proliferate in response to exogenous IL-2 (Fig. 2h). A similar IL-2-induced proliferation of Ndrg1-overexpressing A.E7 cells was observed, comparable to that of TAT–GFP control cells (Fig. 2g). Thus, we conclude that Ndrg1 overexpression induces an anergy-like state.

### Ndrg1 is inactivated through phosphorylation by Akt

**Figure 3:**
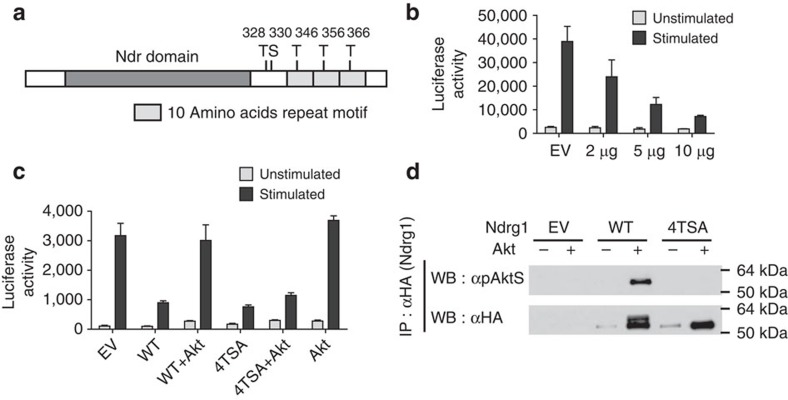
Inhibitory activity of Ndrg1 is abrogated by Akt-dependent phosphorylation. (**a**) Molecular structure of Ndrg1. T, threonine; S, serine. The numbers represent the amino acid location of the threonine or serine residues. The size of each domain or motif is not to scale. (**b**,**c**) Jurkat cells were transfected with Ndrg1 and/or Akt plasmids along with an IL-2 promoter luciferase plasmid. Next, the cells were stimulated with anti-CD3 plus anti-CD28 for 6 h and luciferase activity of cell extracts was measured as described in Methods. (**b**) Dose–response inhibition of IL-2 transcription by Ndrg1 overexpression in Jurkat cells. μg, amount of Ndrg1 plasmid used for transfection; EV, the empty vector. (**c**) Co-transfection of Akt and Ndrg1 plasmids abrogates Ndrg1-mediated inhibition of IL-2 transcription, whereas co-transfection of the 4TSA mutant and Akt does not abrogate 4TSA-mediated inhibition of IL-2 transcription. WT, wild-type Ndrg1; 4TSA, T346A+T356A+T366A+T328A+S330A. (**d**) Akt-dependent phosphorylation of the wild-type Ndrg1 and not the 4TSA mutant. Jurkat cells were transfected with HA-tagged wild-type Ndrg1 or 4TSA mutant plus Akt plasmids. Overexpressed Ndrg1 proteins were immunoprecipitated with anti-HA and immunoblotted with α-pAktS or anti-HA. α-pAktS reacts with phosphorylated serine- or threonine-containing substrate peptides of Akt^26^. IP, immunoprecipitation; WB, western blotting. Results are representative of two (**d**), three (**b**) or four (**c**) independent experiments; error bars, s.d. for quadruplicates of each sample.

According to the two-signal model, anergy factors downstream of TCR signalling should be inhibited by CD28 signalling, to prevent anergy induction. Therefore, we assumed that if Ndrg1 is a mediator of T-cell anergy it should be inactivated by CD28 signalling. Interestingly, the carboxy-terminal region of Ndrg1 has five serine/threonine residues that could be phosphorylated by Akt or its relative Sgk1 (Fig. 3a)^26^,^27^. Akt is known to be a major signalling molecule downstream of CD28 (ref. 28). Hence, we tested whether Ndrg1 could be inactivated by Akt-dependent phosphorylation. For this purpose, we used an IL-2 promoter reporter assay in Jurkat T cells as a readout for the inhibitory activity of Ndrg1. First, we transfected Jurkat cells with both an Ndrg1 expression plasmid and the IL-2 promoter luciferase plasmid, and then stimulated them with anti-CD3 plus anti-CD28. Ndrg1 overexpression efficiently inhibited TCR signal-driven IL-2 transcription in a dose-dependent manner (Fig. 3b), concordant with the TAT–Ndrg1 overexpression studies in T-cell clones and primary T cells. The inhibition of IL-2 transcription by Ndrg1 was abolished when the cells were treated with Phorbol myristate acetate (PMA) plus ionomycin, indicating that Ndrg1 acts at the level of proximal TCR signalling complex (Supplementary Fig. 2). This finding is consistent with the previous observation that anergic T cells proliferate in the presence of PMA plus ionomycin^29^.

Next, we co-transfected the Ndrg1 expression plasmid and an Akt expression plasmid along with the IL-2 promoter luciferase plasmid, to see the effect of overexpression of Akt on the inhibitory activity of Ndrg1. In accordance with our hypothesis, Akt overexpression abrogated Ndrg1's inhibitory activity on IL-2 transcription (Fig. 3c). To see whether Akt inhibited Ndrg1 through direct phosphorylation, we co-transfected Akt and a phosphorylation-defective Ndrg1 mutant, 4TSA, in which all five serine/threonine residues were replaced with alanine. The inhibitory activity of 4TSA could not be abrogated by Akt, in contrast to the wild-type Ndrg1 (Fig. 3c). This was correlated with the inability of the 4TSA mutant to be phosphorylated by Akt, as seen in a western blot analysis of immunoprecipitated Ndrg1 with an anti-phospho-(Ser/Thr) Akt substrate antibody (α-pAktS)^26^ (Fig. 3d). Thus, we conclude that the inhibitory activity of Ndrg1 on TCR signalling can be inactivated by Akt-dependent phosphorylation of the protein.

### Ndrg1 is phosphorylated and degraded by CD28 signalling

**Figure 4:**
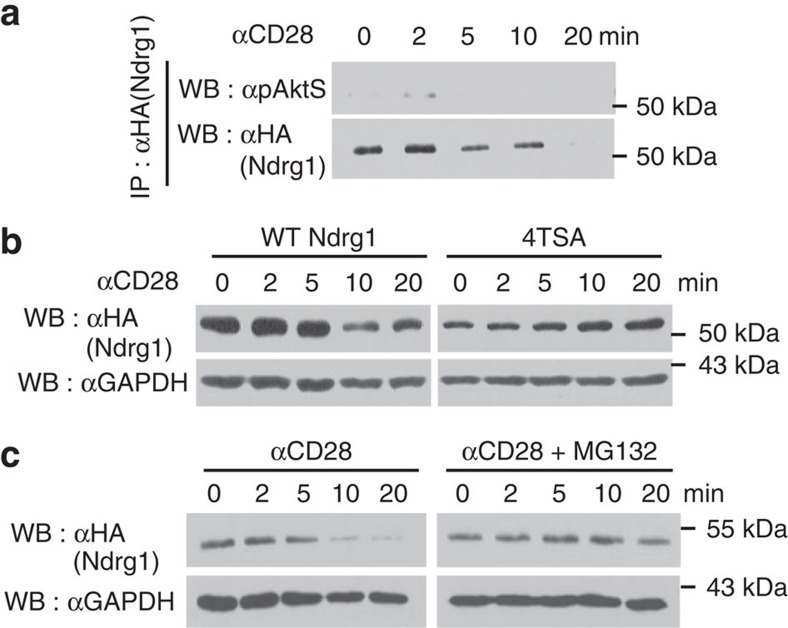
Ndrg1 is phosphorylated and degraded by CD28 signalling. Jurkat cells transfected with HA-tagged wild-type Ndrg1 plasmid (**a**–**c**) or HA-tagged 4TSA mutant plasmid (**b**) were stimulated with anti-CD28 for the indicated times. (**a**) Overexpressed Ndrg1 proteins were immunoprecipitated with anti-HA and immunoblotted with α-pAktS or anti-HA. (**b**,**c**) Cell lysates were immunoblotted with anti-HA or anti-glyceraldehydes 3-phosphate dehydrogenase (GAPDH). Some cells were stimulated with anti-CD28 in the presence of 1 μM of MG132 (**c**). Results are representative of two independent experiments.

Next, we examined whether endogenous CD28 signalling phosphorylates Ndrg1 as well as overexpressed Akt does. We transfected Jurkat cells with an Ndrg1 expression plasmid and delivered the CD28 signal with an agonistic anti-CD28. Phosphorylation of Ndrg1 was detected by western blotting of immunoprecipitated Ndrg1 with α-pAktS. Phosphorylated Ndrg1 was detected as soon as 2 min after stimulation and disappeared thereafter (Fig. 4a). Unexpectedly, the amount of immunoprecipitated Ndrg1 also decreased at those later time points (Fig. 4a). As Ndrg1 phosphorylation preceded Ndrg1 protein disappearance, we suspected that phosphorylated Ndrg1 might be susceptible to protein degradation following CD28 stimulation. To test this idea, we transfected cells with the phosphorylation-defective 4TSA mutant plasmid and stimulated the cells with anti-CD28. The wild-type Ndrg1 protein was greatly diminished by 10 min, whereas the 4TSA mutant protein was not (Fig. 4b). This implies that phosphorylation of Ndrg1 is necessary for its degradation. We then tested to see whether this degradation was proteasome dependent by pretreating the cells with the proteasome inhibitor MG132. Indeed, Ndrg1 degradation was abrogated in the presence of MG132 (Fig. 4c). Therefore, CD28 signalling phosphorylates Ndrg1 and promotes degradation of the protein in a proteasome-dependent manner.

### Phosphorylation of Ndrg1 correlates with anergy blockade

**Figure 5:**
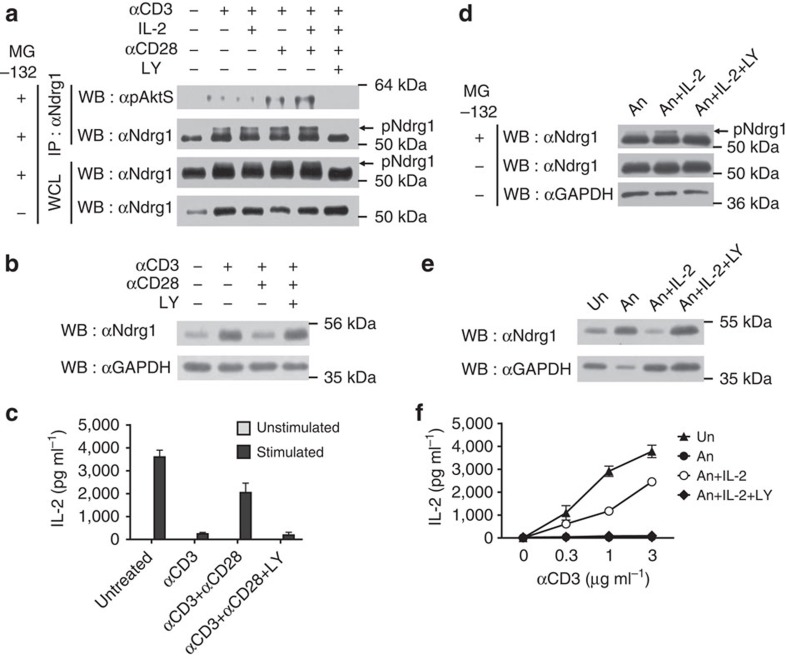
Phosphorylation of Ndrg1 correlates with the blockade of anergy. (**a**) Immunoprecipitated Ndrg1 or the whole-cell lysate (WCL) from A.E7 cells stimulated for 18 h, as indicated in the presence of 1 μM of MG132, was analysed by immunoblotting with α-pAktS or anti-Ndrg1. Some cells were stimulated in the absence of MG132. (**b**,**c**) A.E7 cells stimulated with the indicated reagents for 18 h and rested for additional 5 days were subject to immunoblotting with anti-Ndrg1 (**b**) or restimulating with anti-CD3 plus anti-CD28 to measure IL-2 production (**c**). The cells stimulated with anti-CD3 plus anti-CD28 along with or without LY294002 were rested in the presence of 10 U ml^−1^ of IL-2. (**d**) Immunoblot analysis of Ndrg1 in anergic A.E7 cells treated with IL-2 (1,000 U ml^−1^) or IL-2 plus LY294002 for 24 h in the presence or absence of MG132. Anergic A.E7 cells were generated by 18 h stimulation with anti-CD3 and 5 days resting in the absence of IL-2. (**e**,**f**) Anergic A.E7 cells as in **d** were either left untreated or treated with IL-2 (30 U ml^−1^) for 10 days in the presence or absence of LY294002. Next, the cells were analysed by immunoblotting with anti-Ndrg1 (**e**) or stimulated with various concentrations of anti-CD3 plus anti-CD28 (**f**). LY, LY294002 (25 μM); pNdrg1, phosphorylated Ndrg1; Un, resting untreated (unanergic); An, anergic. Results are representative of two (**c**,**d**) or three (**a**,**b**,**e**,**f**) independent experiments; error bars, s.d.

Based on these data, we checked whether endogenous Ndrg1 becomes phosphorylated and degraded under anergy-blocking conditions. When A.E7 T cells were treated overnight either with anti-CD3 plus anti-CD28 or with those two antibodies along with IL-2 (triple treatment; anti-CD3, anti-CD28 and IL-2), Ndrg1 phosphorylation was significantly enhanced compared with anti-CD3 alone (Fig. 5a). Phospho-Ndrg1 was detected as either α-pAktS-stained immunoprecipitated Ndrg1 or a slower migrating band in anti-Ndrg1 western blotting (Fig. 5a). The identity of the slower migrating band as phospho-Ndrg1 was confirmed by the disappearance of the band on treating the lysate with calf intestinal phosphatase (Supplementary Fig. 3). A similar upwardly shifted band was also observed in Ndrg1/Akt-co-transfected Jurkat cell lysates (Fig. 3d bottom blot and Supplementary Fig. 4). However, the band was not observed in 4TSA/Akt-co-transfected Jurkat cells, further supporting the idea that the shifted band is a phosphorylated form of Ndrg1. Interestingly, these slower migrating bands in A.E7 cells were only observed with cells treated with MG132, indicating that phosphorylated Ndrg1 is normally degraded quickly by the proteasome machinery (Fig. 5a bottom blot). Moreover, phospho-Ndrg1 was not detected when the cells were treated with LY294002, an inhibitor of phosphoinositide 3-kinase (PI3K) that lies upstream of Akt activation. This observation strengthens the idea that phosphorylation of Ndrg1 is mediated by Akt.

Although it seemed clear that CD28 costimulation enhanced Ndrg1 phosphorylation by CD3 stimulation, 18-h-stimulation by anti-CD3 plus anti-CD28 showed only slight, if any, reduction of total Ndrg1 proteins compared with that by anti-CD3 alone (Fig. 5a bottom blot and Supplementary Fig. 5a). CD28 costimulation independency of CD3-mediated Ndrg1 induction was also observed with pre-activated primary CD4^+^ T cells (Supplementary Fig. 5b). However, during the subsequent 5 day-resting period, Ndrg1 protein levels in anti-CD3/anti-CD28-stimulated A.E7 cells decreased to a greater extent than in anti-CD3-stimulated A.E7 cells (Fig. 5b and Supplementary Fig. 6). Accordingly, anti-CD3 plus anti-CD28 treatment followed by 5-day resting led to inhibition of anergy (Fig. 5c). Both Ndrg1 reduction and anergy inhibition by CD28 costimulation could be blocked by the PI3K inhibitor (Fig. 5b,c). Interestingly, CD28 costimulation was not able to reduce Ndrg1 expression during the restimulation phase of anergic cells, which may explain why anergic cells cannot be activated in the presence of CD3/CD28 stimuli and why CD28 stimulation cannot reverse anergic state (Supplementary Fig. 7).

A hallmark of the clonal anergy state is its reversal by IL-2 treatment^30^,^31^. If Ndrg1 is important for anergy maintenance, it should be phosphorylated by IL-2 receptor (IL-2R) signalling and degraded under reversal conditions. When anergic A.E7 cells were treated with IL-2, phosphorylated Ndrg1 was detected in the presence of MG132 and this phosphorylation was inhibited by the PI3K inhibitor (Fig. 5d). This phosphorylation was not detected in the absence of MG132. Finally, when anergy was reversed by IL-2 treatment, the total amount of Ndrg1 was reduced, presumably by proteasome-dependent degradation, which was inhibited by the PI3K inhibitor (Fig. 5e,f). Thus, Ndrg1 plays a critical role in anergy maintenance and IL-2 signalling phosphorylates and degrades Ndrg1 via Akt activation to abrogate the inhibitory activity of Ndrg1.

### Ndrg1 is a downstream target of transcription factor Egr2

**Figure 6:**
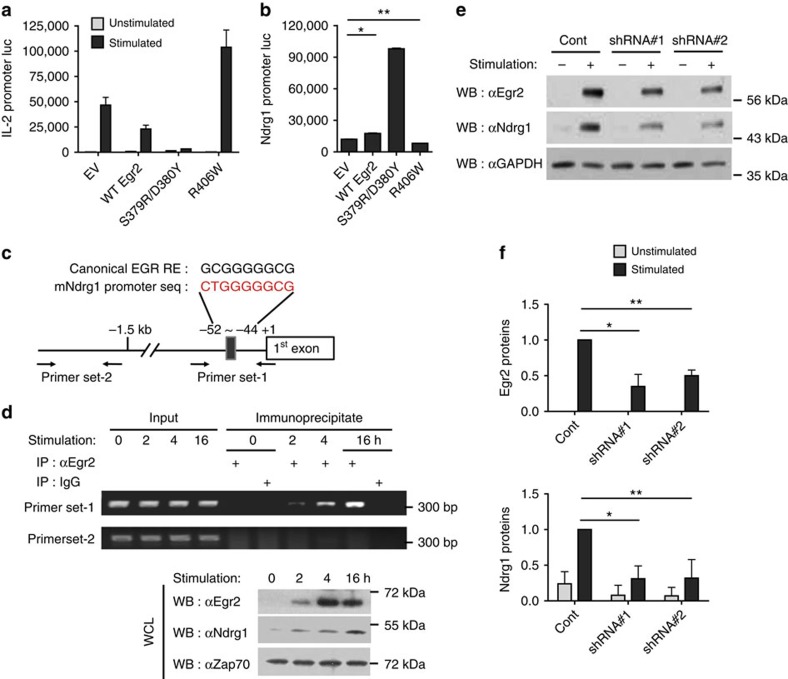
Egr2 binds to the Ndrg1 promoter and enhances Ndrg1 transcription. (**a**,**b**) Jurkat cells were transfected with the wild type (WT) or a mutant Egr2 (S379R/D380Y or R406W) plasmid along with an IL-2 promoter luciferase plasmid (**a**) or an Ndrg1 promoter luciferase plasmid (**b**). The cells were stimulated with anti-CD3 plus anti-CD28 for 6 h in **a**. Luciferase activity of cell extracts was measured as described in Methods. *P=0.0001; **P=0.0002, Student's *t*-test. (**c**) Design of the Ndrg1 promoter primer sets for the ChIP assays in **d**. EGR RE is the EGR response element. (**d**) ChIP assay of Egr2 on Ndrg1 promoter. Co-immunoprecipitated DNA with anti-Egr2 (immunoprecipitate) or total DNA (input) from A.E7 cells stimulated with anti-CD3 for various times, were PCR amplified and subjected to agarose gel electrophoresis (top panel). Immunoblot analysis with anti-Egr2, anti-Ndrg1 or anti-ZAP70 (loading control) from whole-cell lysates (WCL) of A.E7 cells stimulated as above (bottom panel). Results are representative of two (**b**,**d**) or three (**a**) independent experiments. (**e**,**f**) Immunoblot analysis of Egr2 and Ndrg1 on shRNA-mediated knockdown of Egr2. Pre-activated OT-II CD4^+^ T cells transduced with retrovirus encoding control shRNA (Cont) or shRNA to Egr2 (shRNA#1 or shRNA#2) were stimulated with anti-CD3 for 18 h followed by immunoblotting (**e**). Intensity of each band was measured and normalized with glyceraldehydes 3-phosphate dehydrogenase (GAPDH) intensity. The quantified data from three independent experiments are shown in **f**. *and **P<0.05, Student's *t*-test.

The induction kinetics of Egr2 mRNA and proteins was faster than those of Ndrg1 (Fig. 1a and Supplementary Fig. 5a,b). In addition, CD28 costimulation-mediated downregulation of Egr2 proteins clearly paralleled that of Ndrg1 (Supplementary Fig. 6). These observations prompted us to determine whether Egr2 might enhance the transcription of Ndrg1. First, we confirmed the inhibitory effect of Egr2 on T-cell activation by co-transfecting an Egr2 expression plasmid and an IL-2 promoter luciferase plasmid into Jurkat cells followed by anti-CD3/CD28 stimulation. As reported previously^10^, Egr2 moderately inhibited TCR-stimulated IL-2 transcription (Fig. 6a). We also tested the effect of Egr2 mutants on IL-2 transcription. S379R/D380Y and R406W mutants of mouse Egr2 were initially expected to act as dominant-negative mutants similar to their human counterparts (S382R/D383Y found in congenital hypomyelinating neuropathy and R409W in Charcot–Marie–Tooth disease type-1)^32^,^33^. R406W mutant did not show inhibitory effect, but rather potentiated IL-2 transcription as expected. However, surprisingly, S379R/D380Y mutant showed more potent inhibition on IL-2 transcription than the wild-type Egr2, which indicates that this mutant acted as a constitutively active mutant in this case. The functional flexibility of the human counterpart of this mutant (S382R/D383Y) has also been reported in other cell types, such as Schwann cells^34^. Next, to examine the effect of these Egr2 proteins on Ndrg1 transcription, we transfected either Egr2 wild-type plasmid or a mutant plasmid into Jurkat cells along with a mouse Ndrg1 promoter-driven luciferase plasmid. The wild-type Egr2 increased the luciferase activity to a small degree (∼1.5- to 2-fold) (Fig. 6b). On the other hand, S379R/D380Y mutant, a gain-of-function mutant for IL-2 transcription, greatly potentiated luciferase activity (tenfold), which was consistent with its strong inhibitory effect on IL-2 transcription. R406W mutant, a loss-of-function mutant for IL-2 transcription, did not show any increase in luciferase activity but rather showed a slight reduction. Thus, Egr2 appears to induce Ndrg1 transcription, which in turn inhibits IL-2 transcription in the T cells.

Next, we wanted to examine whether this enhancing activity of Egr2 on Ndrg1 transcription was due to direct binding of Egr2 to the Ndrg1 promoter. The proximal region of the Ndrg1 promoter contains a sequence similar to the canonical binding site for Egr family members^35^ (Fig. 6c). We therefore performed a chromatin immunoprecipitation (ChIP) assay for this region in Jurkat cells transfected with the above Ndrg1 promoter plasmid and Egr2 plasmids. The wild-type Egr2 and S379R/D380Y mutant were found to bind to the Ndrg1 promoter, whereas R406W mutant showed a much weaker binding (Supplementary Fig. 8). Binding of S379R/D380Y mutant was stronger than that of the wild-type Egr2, consistent with the results from the luciferase reporter assay. Subsequently, we determined endogenous Egr2 binding to Ndrg1 promoter in the induction phase of anergy in A.E7 cells using ChIP assay. The Egr2 protein was detected bound to the Ndrg1 promoter after the anergic stimulus and the intensity of the interaction gradually increased (Fig. 6d). The Egr2 protein was also gradually increased with a faster kinetics than Ndrg1 protein induction, supporting the idea that Egr2 induces Ndrg1.

Finally, when Egr2 induction was inhibited via anti-Egr2 short hairpin RNAs (shRNAs) in pre-activated CD4^+^ T cells, the induction of Ndrg1 protein was diminished compared with control shRNA treatment (Fig. 6e,f). Thus, Egr2, induced by an anergic stimulus, binds to the Ndrg1 promoter to enhance the expression of Ndrg1.

### Anergy resistance and hyperreactivity of Ndrg1-null T cells

**Figure 7:**
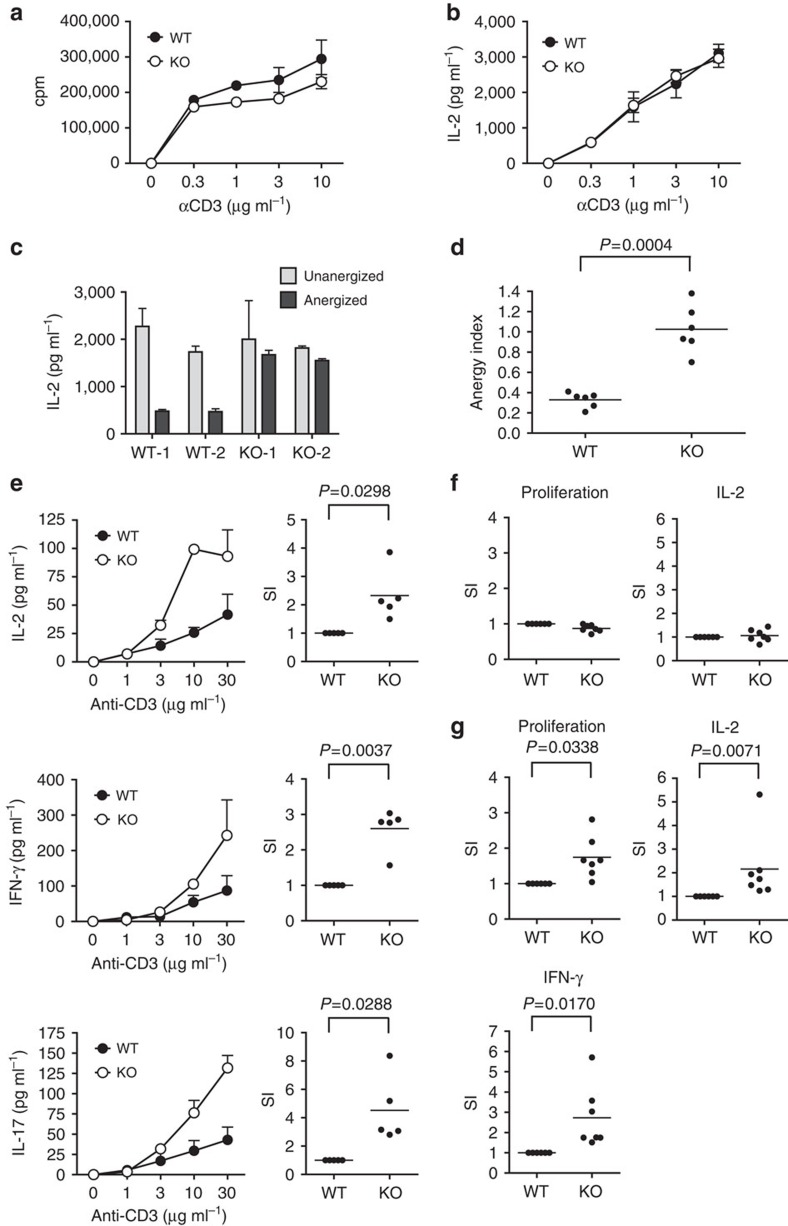
Ndrg1-deficient T cells are resistant to anergy induction and become hyperresponsive in old ages. (**a**,**b**) Lymph node cells from three different mice were isolated and stimulated with various doses of plate-bound anti-CD3 and soluble anti-CD28 (2 μg ml^−1^) for 48–72 h. Cell proliferation (**a**) and IL-2 production (**b**) were measured by ^3^H-thymidine incorporation assay and ELISA assay. The mice were 15 weeks old at the time of analysis. Results are representative of three independent experiments. (**c**) IL-2 production of *in vitro* anergized CD4^+^ T cells from the wild-type (WT) or Ndrg1 knockout (KO) mice on stimulation with anti-TCR plus anti-CD28. Anergy was induced by anti-TCR treatment for 24 h and 3 days resting of *in vitro* pre-activated CD4^+^ T cells. WT-1 or -2 and KO-1 or -2 represent T cells from individual mice. (**d**) Degree of anergy induction in **c** was calculated as an anergy index. Anergy index=IL-2 produced by anergized T cells/IL-2 produced by unanergized T cells. Data were from three separate experiments using two mice per group in each experiment. (**e**) Cytokine production of CD4^+^ T cells isolated from 1.5-year-old wild-type or Ndrg1-knockout mice on stimulation with anti-CD3 plus anti-CD28. Left panel, dose–response curve of cytokine production from a representative set of experiments. Right panel, degree of enhancement of cytokine production calculated as stimulation index (SI). SI, cytokine produced by Ndrg1-knockout T cells/cytokine produced by the wild-type T cells stimulated with 10 μg ml^−1^ (IL-2) or 30 μg ml^−1^ (IFN-γ and IL-17) of anti-CD3 plus anti-CD28. Data were from three independent experiments using five mice per group in total. (**f**,**g**) Proliferation and cytokine production of the naive (**f**) or effector/memory CD4^+^ T cells (**g**) from 1.5-year-old wild-type or Ndrg1-knockout mice on stimulation with anti-CD3 (30 μg ml^−1^) plus anti-CD28 represented as stimulation index (SI). Data were from four independent experiments using six to seven mice per group in total. *P*-values, Student's *t*-test; error bars, s.d.

To provide more definite evidence for Ndrg1's role in T-cell anergy, we carried out loss-of-function experiments using Ndrg1 knockout mice. Immunophenotyping of these mice showed normal thymocyte development and normal distribution of lymphocyte populations in the peripheral lymphoid organs (Supplementary Figs 9 and 10). We first analysed the reactivity of lymph node T cells from 15-week-old Ndrg1 knockout mice, which were mostly naive (CD62L^+^CD44^lo^) at this age (Supplementary Fig. 10f). When these T cells were stimulated with anti-CD3 plus anti-CD28, Ndrg1-deficient T cells proliferated and secreted IL-2 comparably to the wild-type T cells (Fig. 7a,b).

Next, we tested whether Ndrg1-knockout T cells could be anergized *in vitro*. As T-cell clonal anergy is a phenomenon of antigen-experienced previously activated CD4^+^ T cells, we first generated *in vitro* pre-activated T cells by stimulating purified CD4^+^ T cells with anti-TCR plus anti-CD28. Then, these pre-activated T cells were treated with anti-TCR alone to be anergized. When the recovered T cells were restimulated with anti-TCR plus anti-CD28, the wild-type T cells produced much less IL-2 compared with unanergized, pre-activated T cells, which was characteristic of an anergic state. In contrast, anergized Ndrg1-deficient T cells produced equivalent amounts of IL-2 to that from unanergized T cells, meaning that Ndrg1 deficiency in CD4^+^ T cells prevented anergy induction *in vitro* (Fig. 7c,d). We also examined whether the role of Ndrg1 in anergy could be expanded to *in vivo* T-cell anergy, using a peptide-induced *in-vivo* anergy model. When CD4^+^ T cells purified from ovalbumin-specific TCR transgenic (OT-II^+^), *Rag1*^−/−^ mice were adoptively transferred to Thy1.1^+^ congenic mice with subsequent injection of ovalbumin peptide intravenously, the transferred OT-II T cells showed an anergic phenotype with reduced proliferation and IL-2 production compared with unanergized OT-II T cells (Supplementary Fig. 11a,b). By contrast, anergized Ndrg1-deficient OT-II T cells from OT-II^+^, *Ndrg1*^−/−^, *Rag1*^−/−^ mice proliferated comparably to naive Ndrg1-deficient OT-II cells, showing a near-complete resistance to this aspect of anergy induction (Supplementary Fig. 11a,c). However, IL-2 production was only partially restored in the absence of Ndrg1 in these T cells (Supplementary Fig. 11b,d). Thus, peptide-induced anergy was partially prevented by the absence of Ndrg1, implying the differences in molecular mechanisms between clonal anergy and *in vivo* anergy models as proposed previously^36^,^37^. Nonetheless, it is clear that Ndrg1 plays a critical role in some aspects of *in vivo* anergy, such as proliferation blockade. Overall, Ndrg1 deficiency in CD4^+^ T cells brought about failure to induce anergy both *in vitro* and *in vivo*, which suggests Ndrg1 is a critical molecule for anergy induction.

Subsequently, we wanted to find any changes in T-cell reactivity in unmanipulated Ndrg1-deficient mice. In contrast to young Ndrg1-deficient mice that showed no differences in T-cell reactivity (Fig. 7a,b), we were able to detect increased reactivity of T cells in older Ndrg1-deficient mice (1–1.5 years old). When purified CD4^+^ and CD8^+^ T cells from older mice were stimulated *in vitro* with anti-CD3 and anti-CD28, Ndrg1-deficient T cells produced much higher amounts of cytokines, such as IL-2, IFN-γ and IL-17 (Fig. 7e and Supplementary Fig. 12).

One of the phenotypical differences between young and old lymph node T cells was that lymph nodes of older mice had a much higher proportion of effector/memory phenotype T cells (CD62L^−^CD44^hi^) than those of young mice (50–70% versus 10–20%, Supplementary Figs 13a and 10f). Considering the dominant role of Ndrg1 in anergy of T-cell clones, rather than of naive T cells, we postulated that the enhanced responsiveness of old Ndrg1-deficient T cells would be due to hyperreactivity of the effector/memory population. To test this, we purified naive CD4^+^ T cells and effector/memory phenotype T cells from the older Ndrg1-knockout mice and stimulated them separately. As expected, Ndrg1-deficient and wild-type naive CD4^+^ T cells responded comparably (Fig. 7f), whereas Ndrg1-deficient effector/memory phenotype CD4^+^ T cells showed higher proliferation and cytokine production than the wild-type effector/memory phenotype CD4^+^ T cells (Fig. 7g). This preferential effect of effector/memory phenotype T cells was not due to the preferential expansion of this population, because the proportion and number of effector/memory phenotype T cells in older Ndrg1-deficient mice were comparable to those in age-matched wild-type mice (Supplementary Fig. 13a,b). These observations support the idea that Ndrg1 is a clonal anergy-specific mediator and not simply a general negative regulator of TCR signalling.

### Old Ndrg1-deficient T cells can aggravate autoimmunity

**Figure 8:**
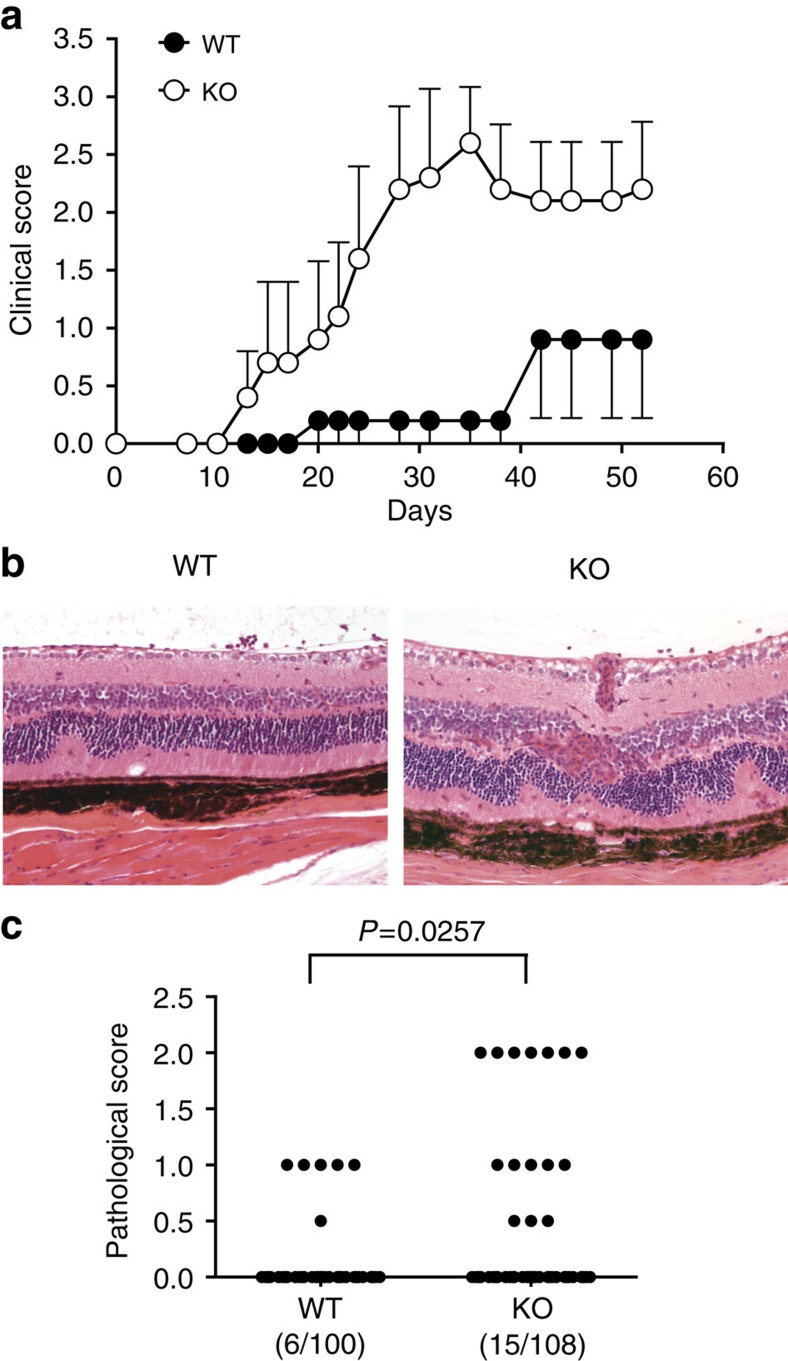
Old Ndrg1-deficient T cells aggravate autoimmunity. (**a**) CD4^+^ T cells isolated from more than 1-year-old wild-type or Ndrg1-knockout mice were adoptively transferred to Rag1^−/−^ mice. EAE was induced and the mean clinical score was measured as described in Methods. Results are representative of two independent experiments; error bars, s.e.m. (**b**,**c**) Experimental autoimmune uveitis (EAU) was induced in 1- to 1.5-year-old wild-type or Ndrg1-knockout mice by injecting them with uveitogenic peptides. The eyes were haematoxylin and eosin stained (**b**) and disease severity was scored histopathologically (**c**). Representative diseased eyes are shown in **b**. The wild-type eye shows focal retinal folds and the knockout eye shows diffuse retinal folds/detachments, vasculitis and granuloma formation. Pooled data from ten independent experiments are shown in **c**. Incidences of diseased eye among total eyes are indicated in parenthesis. *P*-values, Mann–Whitney *U*-test.

Finally, to determine whether these T cells that were hyperresponsive *ex vivo* would have any *in-vivo* effect in a disease model, we adopted a model of myelin oligodendrocyte glycoprotein (MOG) peptide-induced experimental autoimmune encephalomyelitis (EAE) following the adoptive transfer of CD4^+^ T cells^38^. We purified the CD4^+^ T cells from older Ndrg1-knockout mice or B6 controls and adoptively transferred them into lymphopenic *Rag1*^−/−^ mice. Then, EAE was induced by MOG peptide injection in Freund's adjuvant along with pertussis toxin. Indeed, mice receiving Ndrg1-deficient CD4^+^ T cells showed a more severe clinical manifestation of disease (earlier onset and higher score) than the mice with wild-type, age-matched CD4^+^ T cells (Table 1 and Fig. 8a). In this model, the use of *Rag1*^−/−^ mice as the disease hosts allowed us to exclude the encephalitogenic effect of endogenous host T cells and the adoptive transfer of Ndrg1-deficient T cells into Ndrg1-intact *Rag1*^−/−^ hosts precluded the influence from Ndrg1 deficiency in other host tissues. Nonetheless, we further tested whether Ndrg1 deficiency would result in enhanced T-cell-mediated inflammation in older Ndrg1-knockout mice *per se* without adoptive transfer of T cells. For this purpose, we induced experimental autoimmune uveitis by injecting the old Ndrg1-knockout mice with uveitogenic peptides. Despite a low frequency of overall disease induction, Ndrg1-knockout mice showed more severe eye inflammation than the wild-type mice (Fig. 8b,c). On the other hand, when EAE was induced in young Ndrg1-knockout mice, the disease was not more severe than that induced in the age-matched wild-type mice, further confirming that the proinflammatory effect was confined to old Ndrg1-deficient T cells (Supplementary Fig. 14). Consistently, the reactivity of the effector/memory phenotype T cells in young Ndrg1-knockout mice was not enhanced compared with those in young wild-type mice (Supplementary Fig. 15). Thus, old, but not young, Ndrg1-deficient effector/memory phenotype T cells aggravate inducible autoimmune inflammation.

## Discussion

Through intensive studies on the molecular mechanisms of T-cell clonal anergy since its initial discovery more than 20 years ago, the main axis of the induction process has been uncovered. Calcium signalling following TCR stimulation leads to the serial activation and induction of the transcription factors, NFAT and Egr2/3. However, the subsequent effector mechanisms, probably induced by Egr2/3, are still unclear. Most of the molecules claimed to be effector anergy mediators, including Cbl-b, Grail, DGK-α and p27, are involved in negative regulation of naive T cells as demonstrated by the fact that T cells from each knockout mouse showed increased activation of naive T cells^15^,^16^,^39^,^40^. This background enhancement of T-cell activation hampers the interpretation of their role in T-cell clonal anergy, because increased cytokine production and subsequent cytokine-mediated activation of the mammalian target of rapamycin (mTOR), a critical inhibitor of T-cell anergy, can abrogate the anergic state, independent of the direct involvement of these molecules in anergy maintenance^41^. In this context, Egr2 and 3 were the only anergy factors reported up to now that do not show background hyperactivation of naive T cells from genetic ablation^10^,^17^.

Here we demonstrate that Ndrg1 is a novel anergy factor without any effect on naive T-cell reactivity. In the induction phase, newly synthesized Egr2 binds to the Ndrg1 promoter and enhances the expression of Ndrg1. Genetic ablation of murine Ndrg1 made the T cells insensitive to clonal anergy induction. Importantly, the genetic elimination of Ndrg1 did not enhance the background activation of naive T cells (Fig. 7a and Supplementary Fig. 11a,b), eliminating the concern of its indirect involvement in the anergic process. As T-cell clones may represent effector/memory T cells that have experienced antigen multiple times, ‘clonal' anergy may represent the tolerance of effector/memory cells rather than naive cells. In this context, it is interesting to note that old Ndrg1-deficient effector/memory T cells are hyperresponsive, whereas Ndrg1-deficient naive T cells are not (Fig. 7f,g). Thus, Ndrg1 is a clonal anergy-specific mediator operating immediately downstream of Egr2.

It is noteworthy that Ndrg1-knockout naive T cells could be anergized *in vivo* in terms of IL-2 production. Some studies have suggested that *in vitro* clonal anergy and *in vivo* anergy (also called adaptive tolerance) might be different states. Phenotypically, clonally anergic cells showed a preferential block in IL-2 production and the production of other cytokines, including IFN-γ, is less affected in these cells. In contrast, *in-vivo* anergic cells showed more profound defects in the production of most of the cytokines measured^1^. Biochemically, *in-vivo* anergic cells showed a preferential blockade of the calcium pathway over the Ras/mitogen-activated protein kinase pathway, whereas clonally anergic cells showed a more preferential block in the Ras/mitogen-activated protein kinase pathway^36^,^37^. As Ndrg1 seemed to affect *in vitro* anergy more than *in-vivo* anergy, Ndrg1 may provide a biochemical clue for explaining the differences between the two states. Nonetheless, the fact that Ndrg1-knockout T cells could not be partially anergized *in vivo* in terms of proliferation suggests that Ndrg1 may contribute, to some extent, even in the *in-vivo* anergy induction model.

We also explored the role of CD28 costimulation in preventing anergy induction. CD28 could directly deliver an inhibitory signal or instead could increase the production of IL-2 and indirectly inhibit anergy through IL-2R signalling^1^. In either case, the biochemical pathway downstream of both CD28 and IL-2R signalling that is responsible for cell cycle progression and anergy prevention is the PI3K–Akt–mTOR pathway^42^,^43^,^44^. Productive stimulation of CD4^+^ T cells in the presence of the PI3K inhibitor, Wortmannin, or the mTOR inhibitor, rapamycin, induced an anergic state^45^,^46^. The fact that Ndrg1 could be phosphorylated by Akt raised the possibility that Ndrg1 could be regulated by both CD28 costimulation and IL-2R signalling. Indeed, CD28 signalling phosphorylated and degraded Ndrg1 in a proteasome-dependent manner. Ndrg1 was also phosphorylated and degraded by IL-2R signalling under anergy-reversing conditions. Under all conditions, phosphorylation of Ndrg1 could be inhibited by the PI3K inhibitor. Thus, it is very likely to be that Akt-dependent inactivation of Ndrg1 is a common mechanism for antagonizing the anergic state by both CD28 and IL-2R signalling.

The observation that treatment of A.E7 cells with anti-CD3 alone also phosphorylates Ndrg1 to some degree supports the idea that TCR signalling can activate PI3K and Akt even in the absence of CD28 signalling^47^ (Fig. 5a). However, it seems clear that CD28 signalling is more efficient for Akt activation than TCR signalling^43^,^47^. Likewise, the phosphorylation of Ndrg1 by anti-CD3 plus anti-CD28 treatment was more efficient than anti-CD3 treatment alone. Thus, the degree of phosphorylation and elimination of Ndrg1 by Signal 1 versus Signal 1+2 could be a quantitative matter.

Ndrg1 is an inducible cytosolic protein that is expressed ubiquitously in various organs^22^. Although its biological functions are not entirely clear yet, in one report, Ndrg1 mRNA was reduced in cancer cells compared with normal cells and overexpression of Ndrg1 inhibited cell growth^23^. Furthermore, in normal epithelial cells, Ndrg1 mRNA levels peaked at the G1 phase of the cell cycle and decreased as the cells progressed into S phase. T cells in the anergic state show a block in the G1/S transition, and the cell cycle progression of anergic cells stimulated with IL-2 rescues them from anergy^1^,^30^,^46^. Therefore, Ndrg1 may be involved in both cell types in a G1/S transition blockade. However, the fact that Ndrg1 overexpression in Jurkat T cells inhibited TCR-signal-mediated IL-2 transcription indicates that Ndrg1 also inhibits TCR signalling. Consistent with this idea, treatment of Ndrg1-overexpressing Jurkat cells with PMA and ionomycin, which bypasses the proximal signalling complex and delivers downstream activation signals, abrogated the inhibition of IL-2 transcription (Supplementary Fig. 2). Thus, a major site of action of Ndrg1 seems to be at the level of proximal TCR signalling.

Ndrg1-knockout mice showed a relatively mild autoimmune tendency compared with other anergy factor knockout mice such as, Cbl-b^16^ and p27 (ref. 48), because Ndrg1-knockout mice did not show any spontaneous autoimmune inflammation (data not shown). The phenotype of the other mice could be a mixture of naive T-cell hyperactivation and *in-vivo* anergy blockade. As Ndrg1 plays a limited role in anergy induction in naive T cells, effector/memory T-cell hyperresponsiveness in older Ndrg1-knockout mice may represent the real end result of a physiological ‘clonal' anergy blockade: the failure of the tolerance of the effector/memory population. However, whether clonal anergy contributes to tolerance of this population and whether Ndrg1 is a molecular link between these two phenomena needs to be further clarified. Nonetheless, Ndrg1-knockout T cells could clearly enhance inducible autoimmunity, suggesting a role of Ndrg1 in dampening down autoimmunity.

In summary, we propose that Ndrg1 is a new anergy factor downstream of Egr2. In the absence of costimulation, TCR signalling alone upregulates Ndrg1, which inhibits subsequent re-activation of T cells by TCR and CD28 signalling. In the presence of initial costimulation, however, new Ndrg1 is phosphorylated and degraded by Akt via CD28 signalling and/or subsequent IL-2R signalling, which allows the T cells to respond to further TCR and CD28 signalling. In the absence of Ndrg1, effector/memory T cells eventually become hyperresponsive, which can aggravate autoimmunity initiated by other stimuli. This observation may provide an initial glimpse of how clonal anergy can contribute to the prevention of autoimmunity. Further studies on the molecular mechanism of Ndrg1 action and its role in *in-vivo* tolerance will provide a deeper understanding of T-cell clonal anergy.

## Methods

### Mice and cell lines

C57BL6 (B6) and B10.A mice were from SLC, Japan. *Ndrg1*^−/−^ mice on a B6 background (crossbred eight times) were described previously^49^. Ovalbumin-specific TCR(OT-II)-transgenic, *Rag1*^−/−^ mice (B6 background) and pigeon cytochrome *c* (PCC)-specific TCR(5C.C7)-transgenic, *Rag2*^−/−^ mice (B10.A background) were from the NIAID/Taconic Repository (Taconic Farms Inc.). *Rag1*^−/−^ mice (B6 background) and Thy1.1^+^ congenic B6 mice were from the Jackson Laboratory. OT-II^+^, *Ndrg1*^−/−^, *Rag1*^−/−^ mice were generated by cross-breeding *Ndrg1*^−/−^ mice with OT-II^+^, *Rag1*^−/−^ mice. The mice were bred in a specific pathogen-free animal facility at the Research Institute National Cancer Center, Korea and maintained in accordance with the guidelines of the Institutional Animal Care and Use Committee. The A.E7 T cell, a CD4^+^ Th1 clone derived from B10.A mice that recognizes PCC (81-104) specifically, was maintained by repeated stimulation every 2–4 weeks as previously described^25^. Briefly, cells were stimulated with whole PCC protein in the presence of irradiated (3000R) B10.A splenocytes as a source of APC. After 48 h, the cells were expanded in IL-2 (10 U ml^−1^) containing complete medium and rested for at least 2 weeks before restimulation or experimental use. A human T-cell line, Jurkat (clone E6-1), was from ATCC. Pre-activated primary CD4^+^ T cells were generated by stimulating 5C.C7^+^, *Rag2*^−/−^ splenic T cells with PCC peptide (1 μM). After 48 h, the activated T cells were split 1 to 4, expanded in IL-2 (10 U ml^−1^) and complete medium, and rested for at least 14 days before use.

### Antibodies and reagents

Anti-mouse CD3 (145-2C11, #553058), anti-mouse TCR (H57-597, #553167), anti-human CD3 (UCHT1, #555329), anti-human CD28 (CD28.2, #555725), anti-Thy1.2-PE (#553005), anti-TCR Vβ5-FITC (#553189), anti-CD4-APC (#553051), anti-CD44-PE (#553134) and anti-CD62L-FITC (#553150) were from BD Biosciences. Anti-mouse CD28 (37.51) was used as ascites fluid at a final dilution of 1:2,500. The sources of antibodies used for western blottings and immunoprecipitations were as follows: anti-ZAP70 (1:10,000 dilution, #05-776, Upstate Biotechnology), anti-ERK (1:20,000 dilution, #06-182, Upstate Biotechnology), anti-glyceraldehydes 3-phosphate dehydrogenase (1:5,000 dilution, sc-32233, Santa Cruz Biotechnology), anti-phospho-(Ser/Thr) Akt substrate (1:2,000 dilution, #9611, Cell Signaling), anti-haemagglutinin (HA) (1:1,000 dilution, MMS-101P, Covance), anti-Egr2 (1:1,000, PRB-236P, Covance) and anti-actin (1:2,000 dilution, A4700, Sigma). Rabbit anti-Ndrg1 antibody was provided by Thérèse Commes (Université Montpellier, France) or was generated by immunizing rabbits with glutathione *S*-transferase-tagged recombinant murine Ndrg1 protein and subsequent affinity purification (Bethyl Laboratories). Chemicals (Calbiochem) and their final concentrations used for anergy-blocking experiments were as follows: cyclosporine A (1 μM, calcineurin inhibitor), Gö6976 (5 μM, protein kinase C inhibitor), PD98059 (50 μM, Erk inhibitor), SB203580 (5 μM, p38 inhibitor) and rapamycin (1 μM, mTOR inhibitor). MG132, LY294002 and PCC proteins were from Sigma. Mouse recombinant IL-2 was from Biosource International. Ovalbumin (323–339) (ISQAVHAAHAEINEAGR), murine MOG (35–55) (MEVGWYRSPFSRVVHLYRNGK) and human IRBP (1–20) (GPTHLFQPSLVLDMAKVLLD) peptides were from Peptron.

### *In vitro* clonal anergy

A.E7 cells (2.5 × 10^6^ ml^−1^) were anergized by stimulation with 1–10 μg ml^−1^ of plate-bound anti-TCR (H57-597) or anti-CD3 (145-2C11) for 16 h. After washing, the cells were ‘rested' in the absence of IL-2 for 5 days before use. For *in vitro* anergy of *Ndrg1*^−/−^ T cells, CD4^+^ T cells (2.5 × 10^6^ ml^−1^) purified from the spleen and lymph nodes using anti-CD4-microbeads (Miltenyi Biotec) were pre-activated by stimulation with plate-bound anti-TCR(1 μg ml^−1^) and anti-CD28 (37.51) for 48 h followed by 3 days resting in the presence of IL-2 (10 U ml^−1^). Pre-activated T cells (2.5 × 10^6^ ml^−1^) were anergized by stimulation with 3 μg ml^−1^ of plate-bound anti-TCR for 24 h and 3 days resting in the absence of IL-2. The anergic state was evaluated by measuring the IL-2 produced by anergic cells (1 × 10^5^ ml^−1^) on restimulation with anti-CD3 (1 μg ml^−1^) and anti-CD28 for 24 h (A.E7) or anti-TCR (1 μg ml^−1^) and anti-CD28 for 48 h (pre-activated T cells).

### DNA microarray

RNAs isolated from A.E7 cells were reverse transcribed to make complementary DNAs using a T7-oligo-dT primer. Biotinylated cRNA probes were prepared from a T7 RNA polymerase-mediated *in vitro* transcription reaction. The probes were hybridized to Affymetrix mouse MU74A, B and C gene chips and stained with streptavidin–phycoerythrin. Hybridization and analysis of the fluorescent intensities were done at the NIAID core facility according to Affymetrix protocols (Affymetrix Microarray Suite User Guide, 1999, Affymetrix). A microarray chip has 16 different oligonucleotides (perfect match) per gene to represent a single gene. For each oligonucleotide, a control oligonucleotide with a single base pair mismatch (mismatch) was used for the subtraction of background signal. ‘Average difference' represents the difference in the fluorescence intensity between perfect match and mismatch. Expression data were processed and normalized using Affymetrix Microarray Suite, Version 4.0 (Affymetrix). Data used for the analysis of the first set of microarray (drug treatment) were from GEO database (accession number GSE2323; ref. 10). For the second set of microarray (kinetic analysis), the data for the whole genes are deposited to GEO database (accession number GSE72731).

### Plasmid constructs

Mouse Ndrg1 cDNA was amplified by RT–PCR from stimulated A.E7 cells and subcloned in-frame into an HA-tagged pHM6 expression plasmid (Roche) for transfection and into pTAT-HA (a gift from Steve Dowdy, UCSD, CA) for TAT-fusion protein production. The Akt and Egr2 expression plasmids were generated by cloning murine Akt1 cDNA and murine Egr2 cDNA (ImaGenes) into an EF1α promoter-driven expression vector (pcEFL, a gift from L. Samelson, NCI/NIH, MD), to assure efficient expression in T cells. Point mutants of Ndrg1 in pHM6 and Egr2 in pcEFL were generated by QuikChange site-directed mutagenesis (Stratagene). The Ndrg1 promoter luciferase plasmid was made by cloning the PCR-amplified murine Ndrg1 promoter (−637 to approximately +76) into a pGL3-Basic vector (Promega). All the sequences were confirmed by automated DNA sequencing.

### TAT-fusion potein transduction

Expression and purification of recombinant TAT-fusion proteins from the BL21 (DE3) pLysS strain of *Escherichia coli* were performed as described^25^. A.E7 cells were washed twice with PBS and incubated with TAT–Ndrg1 or TAT–GFP (100 nM) for 2 h in a CO_2_ incubator before stimulation.

### Cell proliferation assay and cytokine ELISA

Cells (1 × 10^5^ ml^−1^) were stimulated with various concentrations of plate-bound anti-CD3 plus anti-CD28. After 48 h, one half of the culture supernatant was collected to measure cytokines and the remaining cells and medium were pulsed with 1 μCi of ^3^H-thymidine per well for an additional 24 h. Cells were harvested using a Tomtec 96-well harvester and the radioactivity was counted in a Wallac Trilux 1450 scintillation counter. Secreted cytokines were measured using ELISA kits (R&D Systems) or ELISA sets (BD Biosciences, eBioscience) according to the manufacturer's instructions.

### Transient transfection and the luciferase assay

Jurkat T cells (1.0–2.0 × 10^7^), mixed with an HA–Ndrg1 or Egr2 expression plasmid, human IL-2 promoter firefly luciferase plasmid (a gift from S. Hoffmann, NIDDK/NIH, MD) and a pRL-TK *Renilla* luciferase control plasmid for normalization (Promega), were electroporated at 250 V and 950 μF in a 0.4-cm-gap cuvette using Gene Pulser (Bio-Rad Laboratories) and allowed to recover for 24 h before stimulation. Mouse IL-2 promoter luciferase plasmid or mouse Ndrg1 promoter luciferase plasmid was used in place of human IL-2 luciferase plasmid in some experiments.

For stimulation, a 96-well plate coated with goat anti-mouse IgG (10 μg ml^−1^, #115-005-003, Jackson Immunoresearch) overnight was washed and coated with anti-CD3 (UCHT1, 1 μg ml^−1^) for 2 h at room temperature. Cells (1 × 10^5^) were added to each well with soluble anti-CD28 (CD28.2, 2 μg ml^−1^) and incubated at 37 °C for 6 h followed by lysis. Luciferase activity was measured with a luminometer (VICTORlight, Perkin Elmer) using a Dual-luciferase reporter assay system according to the manufacturer's instructions (Promega). Firefly luciferase activity was normalized to *Renilla* luciferase activity.

For western blotting and immunoprecipitation experiments, HA–Ndrg1 and/or Akt plasmids without luciferase plasmids were used in the above transfection protocol. For anti-CD28 stimulation of HA–Ndrg1-transfected cells, the cells were treated with anti-CD28 (CD28.2, 2 μg ml^−1^) for 10 min on ice, followed by cross-linking with goat anti-mouse IgG (5 μg ml^−1^, #115-005-003, Jackson Immunoresearch) for 10 min on ice. Next, cells were placed in a 37 °C water bath for the indicated time and the reaction was stopped by adding ice-cold PBS.

### Immunoprecipitation and western blotting

For immunoprecipitations, cell lysates were incubated with anti-HA (2 μg, MMS-101P, Covance) for 3 h at 4 °C, followed by incubation with 30 μl of protein G-agarose beads (Santa Cruz Biotechnology) for 1 h. Immune complexes were washed five times in lysis buffer before western blotting. For western blotting, the immunoprecipitated proteins or total cell lysates were subjected to SDS–PAGE, transferred to a Nitrocellulose membrane (Millipore) and probed with the indicated antibodies. Horseradish peroxidase (HRP)-conjugated secondary antibodies (anti-mouse IgG-HRP, 1:10,000 dilution, #115-035-003; anti-rabbit IgG-HRP, 1:10,000 dilution, #111-035-003; and anti-rabbit IgG-HRP, light chain-specific, 1:10,000 dilution, #211-032-171; Jackson Immunoresearch Laboratories) were used to detect primary antibodies. Blottings were visualized by a chemiluminescence reaction using SuperSignal West Pico (Pierce). Images for Figs 1b–d, 3d, 4a–c, 5a,b,d,e and 6d,e, and Supplementary Figs 3 and 5–7 have been cropped for presentation. Full-size images are presented in Supplementary Fig. 16.

### ChIP assay

ChIP assays were performed using an EZ ChIP kit (Millipore) according to the manufacturer's instructions. A.E7 cells (5 × 10^6^) stimulated with anti-CD3 (1 μg ml^−1^) for the indicated times were cross-linked with 1% formaldehyde for 10 min at room temperature. After quenching of the formaldehyde with 125 mM glycine, the chromatin was sheared by sonication. Fragmented chromatin was pre-cleared for 1 h with BSA-blocked protein G beads and incubated with anti-Egr2 (3 μg, PRB-236P, Covance) or normal rabbit IgG(3 μg, #sc-2027, SantaCruz Biotechnology) at 4 °C overnight. Immunocomplexes were precipitated with blocked protein G beads. DNA was recovered by reverse cross-linking, purified and used as a template for PCR with primers for the Ndrg1 promoter. Primer set-1, sense: 5′-GGG CAC ACG TTC GCT GCA CA-3′; antisense: 5′-CTG GCA AGG TTT GTT TAT GTC-3′ (the amplicon includes sequence from +16 to −285 of murine Ndrg1 promoter). Primer set-2, sense: 5′-GCA GCA GAA AAT TCA ATG CT-3′; antisense: 5′-TCT GTT ACC CCA TTA ATA AC-3′ (the amplicon includes sequence from −1,865 to −1,527 of murine Ndrg1 promoter).

### shRNA-expressing retroviral transduction

shRNA oligonucleotides against mouse Egr2 or LacZ (as a control) were cloned into shRNA-expressing retroviral vector pRetroSuper-GFP as previously described^50^. First, pRetrosuper-Puro plasmids containing shRNA oligos against Egr2 and LacZ were generated by digestion of the pRetrosuper-Puro vector with BglII and HindIII, and ligation with the annealed oligos. shRNA oligonucleotide sequences are as follows (target sequences indicated in capitals). Egr2-shRNA#1-sense: 5′-gatccccGTGACCACCTTACTACTCAttcaagagaTGAGTAGTAAGGTGGTCAC tttttggaaa-3′, Egr2-shRNA#1-antisense: 5′-agcttttccaaaaaGTGACCACCTTACTACTCAtctcttgaaTGAGTAGTAAGGTGGTCACggg-3′; Egr2-shRNA#2-sense: 5′-gatccccGTTTGCCAGGAGTGACGAAttcaagagaTTCGTCACTCCTGGCAAACtttttggaaa-3′, Egr2-shRNA#2-antisense: 5′-agcttttccaaaaaGTTTGCCAGGAGTGACGAAtctcttgaaTTCGTCACTCCTGGCAAACggg-3′; and LacZ-shRNA-sense: 5′-gatccccGTGACCAGCGAATACCTGTttcaagagaACAGGTATTCGCTGGTCACtttttggaaa-3′, LacZ-shRNA-antisense: 5′-agcttttccaaaaaGTGACCAGCGAATACCTGTtctcttgaaACAGGTATTCGCTGGTCACggg-3′. Next, the AgeI/XhoI fragments from shRNA oligo-containing pRetrosuper-Puro plasmids were cloned into the AgeI/XhoI site of pRetrosuper-GFP Smad3 (a gift of Joan Massague, Addgene plasmid #15723) replacing pre-existing Smad3 shRNA, by which pRetrosuper-GFP plasmids containing anti-Egr2 and anti-LacZ shRNA oligos were constructed.

Ecotropic retroviruses were produced using the PhoenixEco packaging cell line as previously described^51^. Briefly, the retroviral plasmids and a plasmid encoding VSV-G cDNA (pMD.G) were transiently transfected into Phoenix GP cell line using Lipofectamine 2000 (Invitrogen). After 48 h, the culture supernatant containing VSV-G pseudotyped retrovirus was harvested. Next, Phoenix Eco cell line was transduced with the retrovirus-containing supernatant for overnight. After 3–5 days, GFP-positive Phoenix Eco cells were purified by a cell sorter (FACS Aria, BD Biosciences), to generate stable cell lines for producing ecotropic retroviruses. The culture supernatant containing ecotropic retrovirus was harvested and then concentrated tenfold using a centrifugal filter device (Amicon Ultra-15, 100 kDa cutoff, Millipore) for murine T-cell transduction. For retroviral transduction, splenic OT-II T cells from OT-II^+^, *Rag1*^−/−^ mice were stimulated by ovalbumin peptides. Twenty-four hours after stimulation, T cells were transduced with the retroviruses by centrifuging the cells at 1,250 g for 90 min (spin infection) in the presence of 6 μg ml^−1^ Polybrene (Sigma-Aldrich). This procedure was repeated once on the same day. Seventy-two hours after stimulation, the transduced T cells were transferred to a fresh medium containing 10 U ml^−1^ mouse IL-2 (Invitrogen) and ‘rested' for 7days without further stimulation before analysis.

### *In-vivo* anergy model

OT-II T cells (1–1.2 × 10^7^) purified from OT-II^+^, Thy1.2^+^, *Rag1*^−/−^ or OT-II^+^, Thy1.2^+^, *Rag1*^−/−^, *Ndrg1*^−/−^ mice using anti-CD4-microbeads (Miltenyi Biotec) were intravenously injected into Thy1.1^+^ congenic B6 mice. The next day, 500 μg of ovalbumin peptide was injected intravenously. After 5 days, CD4^+^ T cells were purified from the spleen and lymph nodes of the recipient mice using a CD4-negative selection kit (Miltenyi Biotec). The percentage of OT-II T cells (TCR Vβ5^+^ and Thy1.2^+^) was calculated by flow cytometry (anti-TCR Vβ5-FITC, 1:100 dilution and anti-Thy1.2-PE, 1:1,600 dilution). OT-II T cells (4 × 10^4^) were stimulated with various concentrations of ovalbumin peptide in the presence of irradiated B6 splenocytes (4 × 10^5^) for 48–72 h. Purified uninjected OT-II T cells were used as negative controls. Cell proliferation and IL-2 production were measured as described above.

### T-cell purification from the old mice

For stimulation of T cells from 1.5-year-old *Ndrg1*^−/−^ mice or age- and sex-matched normal B6 mice, CD4^+^ and CD8^+^ T cells were purified using anti-CD4 microbeads and anti-CD8 microbeads (Miltenyi Biotec). For purification of naive and effector/memory CD4^+^ T cells, CD4^+^ T cells purified using the CD4-negative selection kit were stained with anti-CD4-APC (1:800 dilution), anti-CD44-PE (1:1,000 dilution) and anti-CD62L-FITC (1:200 dilution). The CD4^+^CD44^low^CD62L^high^ population (naive T cells) and the CD4^+^CD44^high^CD62L^low^ population (effector/memory T cells) were further purified using a cell sorter (FACS Aria, BD Biosciences) to >98% purity.

### Experimental autoimmune encephalomyelitis (EAE) model

CD4^+^ T cells (5 × 10^6^) purified using anti-CD4 microbeads from 1- to 1.5-year-old *Ndrg1*^−/−^ mice or age- and sex-matched normal B6 mice were intravenously injected into *Rag1*^−/−^ mice. EAE was induced by subcutaneous injection of 300 μg MOG (35–55) peptide in CFA (Pierce) with 500 μg *Mycobacterium tuberculosis* strain H37RA on days 1 and 8, supplemented by intravenous injections of 200 ng pertussis toxin (Sigma) on days 1 and 3. The mice were observed every other day for clinical signs and scored on a scale of 0–5 with gradations of 0.5 for intermediate scores: 0, no clinical signs; 1, loss of tail tone; 2, wobbly gait; 3, hindlimb paralysis; 4, hind and forelimb paralysis; 5, death. Five or six recipient mice per group were used for each experiment.

### Experimental autoimmune uveitis model

One to 1.5-year-old *Ndrg1*^−/−^ mice or age- and sex-matched normal B6 mice were immunized subcutaneously in the upper neck region with 250 μg of IRBP peptide in PBS/IFA (1:1, vol/vol, Sigma) supplemented with 250–500 μg of *M. tuberculosis* strain H37RA (Difco). Concurrently, 500–1,000 ng of pertussis toxin (Sigma) was injected intraperitoneally as an additional adjuvant. The eyes were enucleated on day 21, prefixed in 4% glutaraldehyde (Sigma) in PBS for 1 h and then transferred to 10% formaldehyde (Sigma) in PBS. Fixed and dehydrated eyes were embedded in paraffin blocks and the sections were stained with haematoxylin and eosin. The disease severity was scored histopathologically on a scale of 0–4: 0, no change; 0.5, mild inflammatory cell infiltration, no damage; 1, mild inflammatory cell infiltration, retinal folds and focal retinal detachments, few small granulomas, perivascularitis; 2, moderate infiltration, retinal folds and detachments, focal photoreceptor cell damage, small- to medium-sized granulomas, perivascularitis and vascularitis; 3, medium to heavy infiltration, extensive retinal folding with detachments, moderate photoreceptor cell damage, medium-sized granulomatous lesions, subretinal neovascularization; 4, heavy infiltration, diffuse retinal detachment with serous exudate and subretinal bleeding, extensive photoreceptor cell damage, large granulomatous lesions and subretinal neovascularization.

## 

## Additional information

**How to cite this article:** Oh, Y. M. *et al.* Ndrg1 is a T-cell clonal anergy factor negatively regulated by CD28 costimulation and interleukin-2. *Nat. Commun.* 6:8698 doi: 10.1038/ncomms9698 (2015).

## Supplementary Material

Supplementary Information Supplementary Figures 1-16 and Supplementary Table 1

## Figures and Tables

**Table 1:** Clinical parameters of EAE after adoptive transfer of old CD4^+^ T cells.

**Experimental group**	**Disease incidence**	**Disease onset (mean±s.e.m.)**	**Maximum score (mean±s.e.m.)**
WT T-cell transfer	6/11 (54.5%)	29.0±3.8	1.41±0.49
KO T-cell transfer	10/11 (90.9%)	20.0±2.6	2.95±0.44*

Data are pooled from two independent experiments, each with 5 or 6 mice per experimental cohort (total 11 mice per group). *P<0.05 (Mann–Whitney test).
