# Supplementary material for: Ndrg1 is a T-cell clonal anergy factor negatively regulated by CD28 costimulation and interleukin-2
Source: Nat Commun. 2015 Oct 28;6:8698. doi: 10.1038/ncomms9698 (PMC4846325; doi:10.1038/ncomms9698)
Supplement: Supplementary Information — Supplementary Figures 1-16 and Supplementary Table 1 [file ncomms9698-s1.pdf]

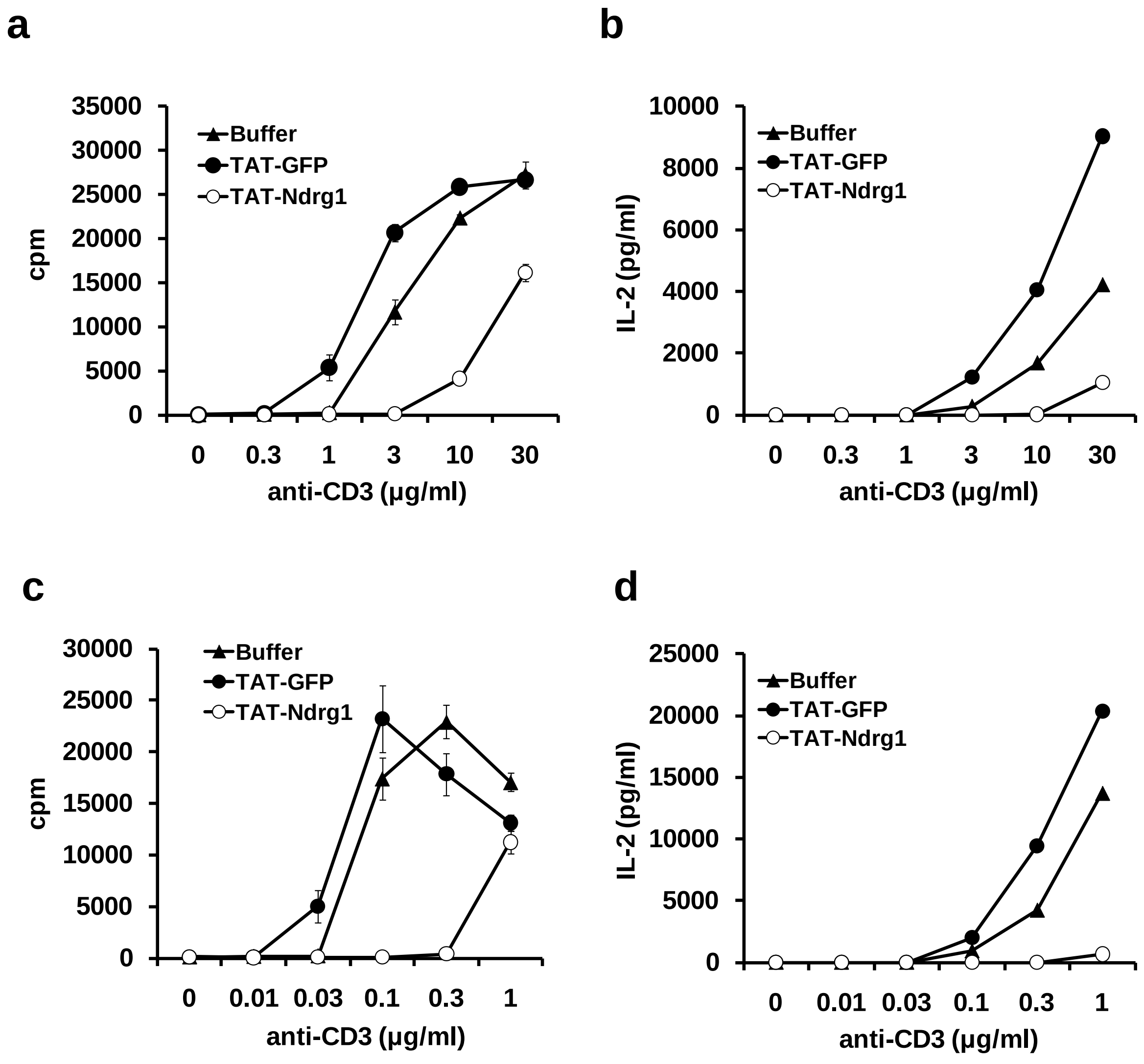

Supplementary Figure 1. Ndrg1 overexpression inhibits activation of naïve T cells and pre-activated T cells. Proliferation(**a,c**) or IL-2 production(**b,d**) of TAT-protein transduced naïve $CD4^+$ T cells (**a,b**) or pre-activated $CD4^+$ T cells (**c,d**) stimulated with various concentrations of anti-CD3 plus anti-CD28 for 48h, was measured by ^3H-thymidine incorporation assay or ELISA. Naïve CD4 T cells were isolated from lymph nodes of pigeon cytochrome C(PCC)-specific TCR transgenic(5C.C7), $Rag2^{-/-}$ mice. Preactivated T cells were generated by 48hrs of stimulation of splenocytes from $5CC7^+$, $Rag2^{-/-}$ mice with antigenic PCC peptide followed by 14d resting in the presence of 10U ml^{-1} of IL-2.

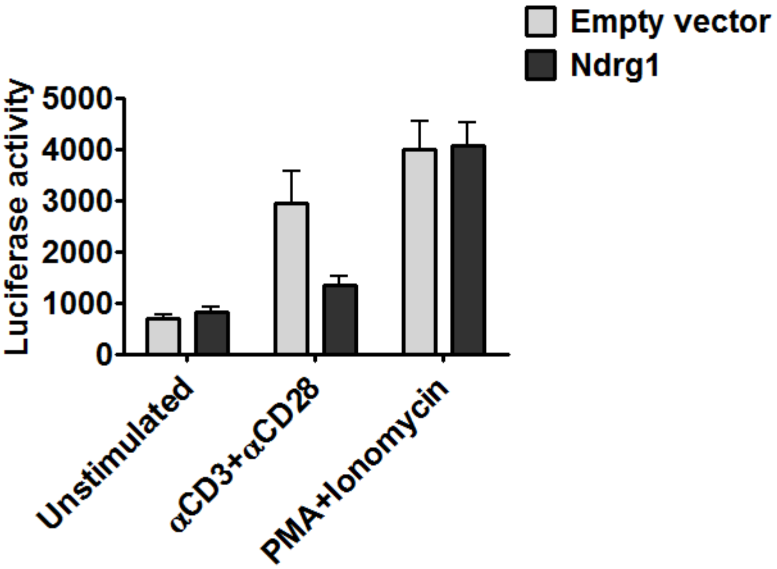

Supplementary Figure 2. PMA plus ionomycin treatment of Ndrg1-overexpressing Jurkat cells abrogates the inhibitory activity of Ndrg1. Jurkat cells were transfected with Ndrg1 or empty vector plasmids along with an IL-2 promoter luciferase plasmid. Then, the cells were stimulated either with anti-CD3 (10μg ml^{-1}) plus anti-CD28 (10μg ml^{-1}) or with PMA(20ng ml^{-1}) plus ionomycin (20mM) for 6h and luciferase activity of cell extracts was measured as described in materials and methods. (error bars, s.d. for quadruplicates of each sample)

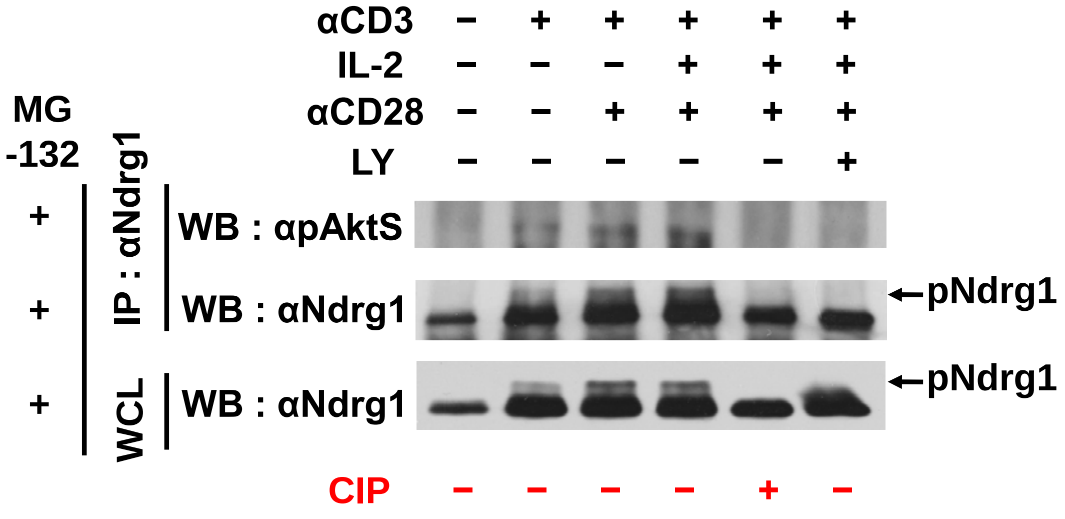

Supplementary Figure 3. Verification of the identity of phospho-Ndrg1 bands by phosphatase treatment. Immunoprecipitated Ndrg1, or the whole cell lysate (WCL), from A.E7 cells stimulated for 18h as indicated in the presence of 1μM of MG132, was incubated at 37°C for 1h in the presence or absence of 5U of calf intestinal phosphatase (CIP), and then analyzed by immunoblotting with α-pAktS or anti-Ndrg1.

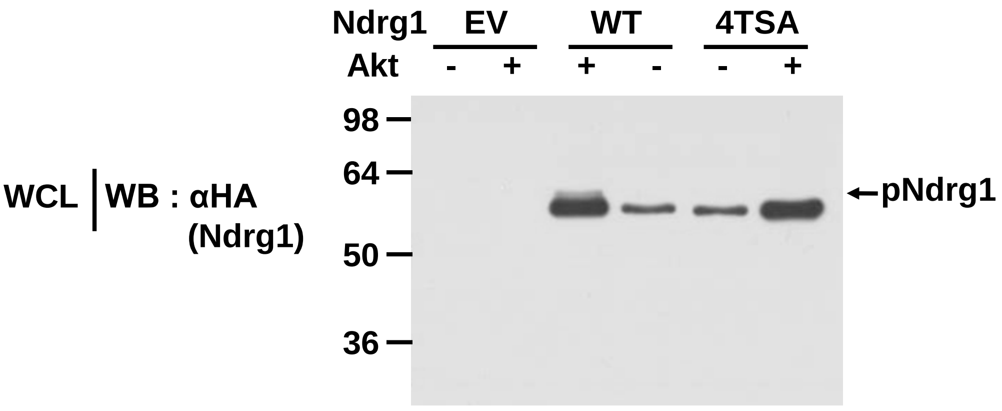

Supplementary Figure 4. Appearance of phospho-Ndrg1 band is Akt-dependent in Jurkat cells. Jurkat cells were transfected with HA-tagged wild type Ndrg1 or 4TSA mutant plus Akt plasmids. The whole cell lysate (WCL) was subjected to immunoblotting with anti-HA. EV, the empty vector; WT, the wild type Ndrg1; WB, western blot.

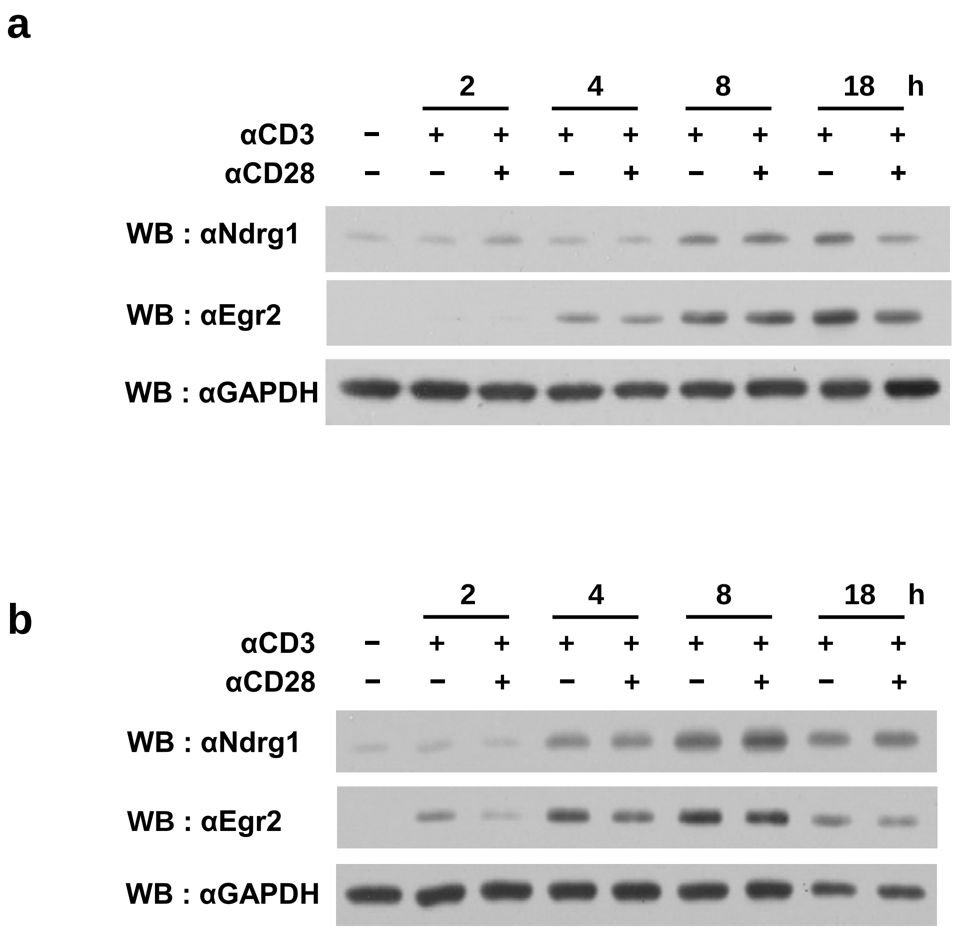

Supplementary Figure 5. Effect of CD28 costimulation on CD3-mediated induction of Ndrg1 and Egr2 in the anergy induction phase. Resting A.E7 cells (**a**) or pre-activated primary CD4 T cells (**b**) were stimulated with anti-CD3 or anti-CD3 plus anti-CD28 for the indicated times. Cell lysates were analyzed by immunoblotting with anti-Ndrg1 or anti-Egr2. GAPDH was used as a loading control for the immunoblots. Pre-activated CD4 T cells were generated by stimulating splenocytes from OTII TCR transgenic mice with ovalbumin peptide for 3d followed by 7d resting in the presence of IL-2 (10U ml^{-1}).

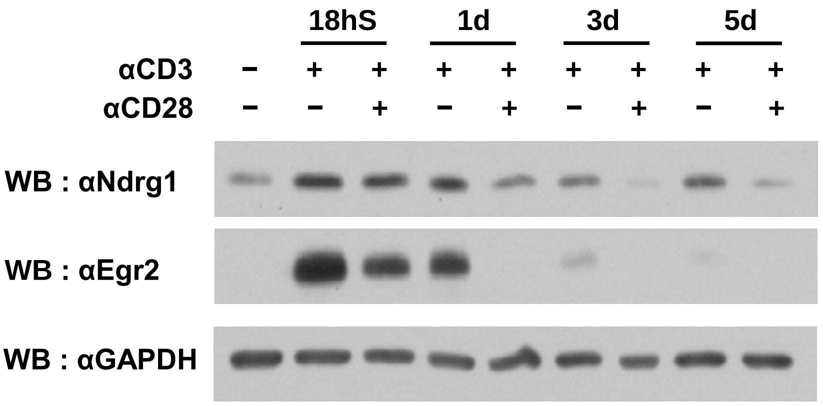

Supplementary Figure 6. Effect of CD28 costimulation on the downregulation of Ndrg1 and Egr2 during the resting period following anergy induction. Resting A.E7 cells stimulated with anti-CD3 or anti-CD3 plus anti-CD28 for 18h were either harvested for immunoblotting (18hS) or rested for 1d, 3d or 5d. Cell lysates from various time points were analyzed by immunoblotting with anti-Ndrg1 or anti-Egr2. GAPDH was used as a loading control for the immunoblots. The cells stimulated with anti-CD3 plus anti-CD28 were rested in the presence of 10U ml^{-1} of IL-2.

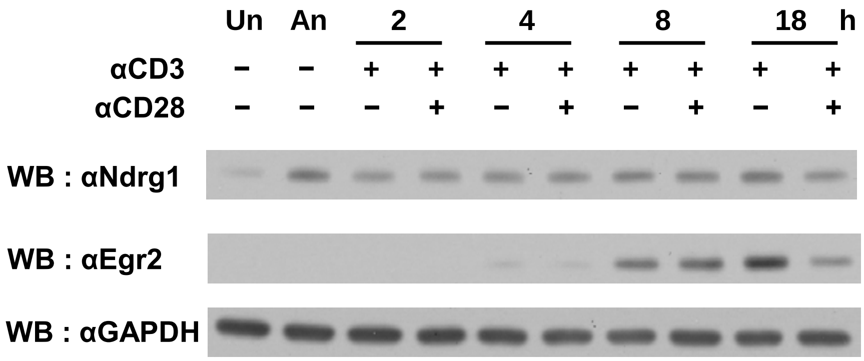

Supplementary Figure 7. Effect of CD28 costimulation on the expression levels of Ndrg1 and Egr2 in the restimulation phase of anergic cells. Anergized A.E7 cells were stimulated with anti-CD3 or anti-CD3 plus anti-CD28 for the indicated times. Cell lysates were analyzed by immunoblotting with anti-Ndrg1 or anti-Egr2. GAPDH was used as a loading control for the immunoblots. Anergized cells were generated by stimulating A.E7 cells with anti-CD3 for 18h followed by 5d resting in the absence of IL-2. Un, resting unanergized; An, anergized.

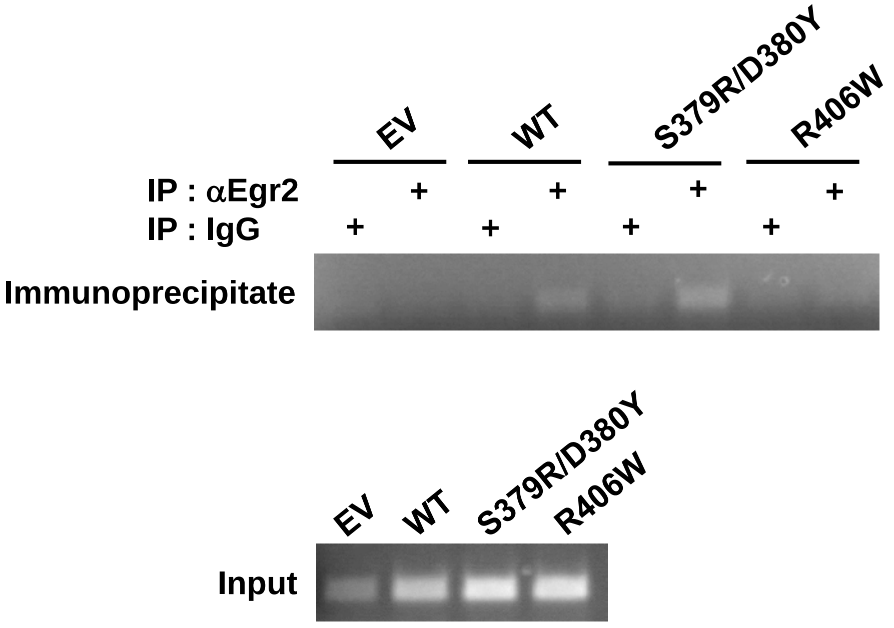

Supplementary Figure 8. Analysis of direct binding of Egr2 wild type and mutants to the Ndrg1 promoter. Jurkat cells were transfected with the wild type (WT) or a mutant Egr2 (S379R/D380Y or R406W) plasmid along with an Ndrg1 promoter luciferase plasmid. After 24h, ChIP assay of Egr2 on Ndrg1 promoter was performed. Co-immunoprecipitated DNA with anti-Egr2 or control IgG (Immunoprecipitate) or total DNA (Input) from the Jurkat cells, were PCR-amplified with primer set-1 in Fig 6c and subjected to agarose gel electrophoresis.

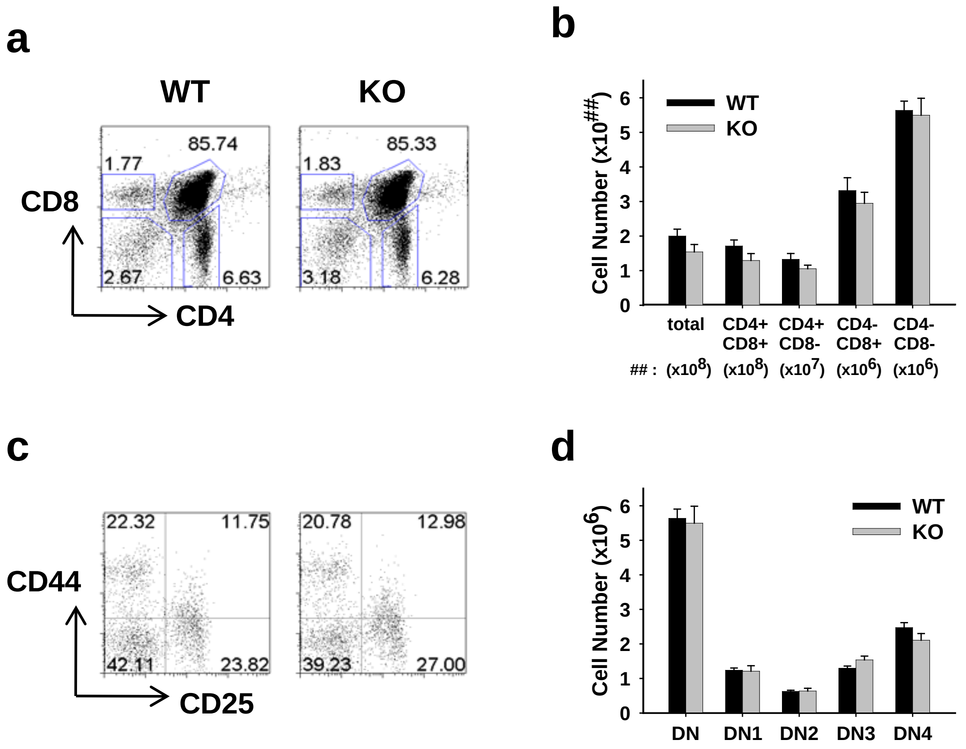

Supplementary Figure 9. Normal thymic T cell development in Ndrg1 knockout mice. Thymocyte profile by flow cytometry analysis of total thymocytes (**a,b**) or $CD4^{-}CD8^{-}$ double negative population (**c,d**). The percentages of cells of each subpopulation were displayed in the representative dot blots (**a,c**) or the absolute cell number of each subpopulation of 4 different mice was averaged (**b,d**). DN1, $CD44^{+}CD25^{-}$; DN2, $CD44^{+}CD25^{+}$; DN3, $CD44^{-}CD25^{+}$; DN4, $CD44^{-}CD25^{-}$. The mice were 14-15 weeks old at the time of analysis. (error bars, s.e.m.)

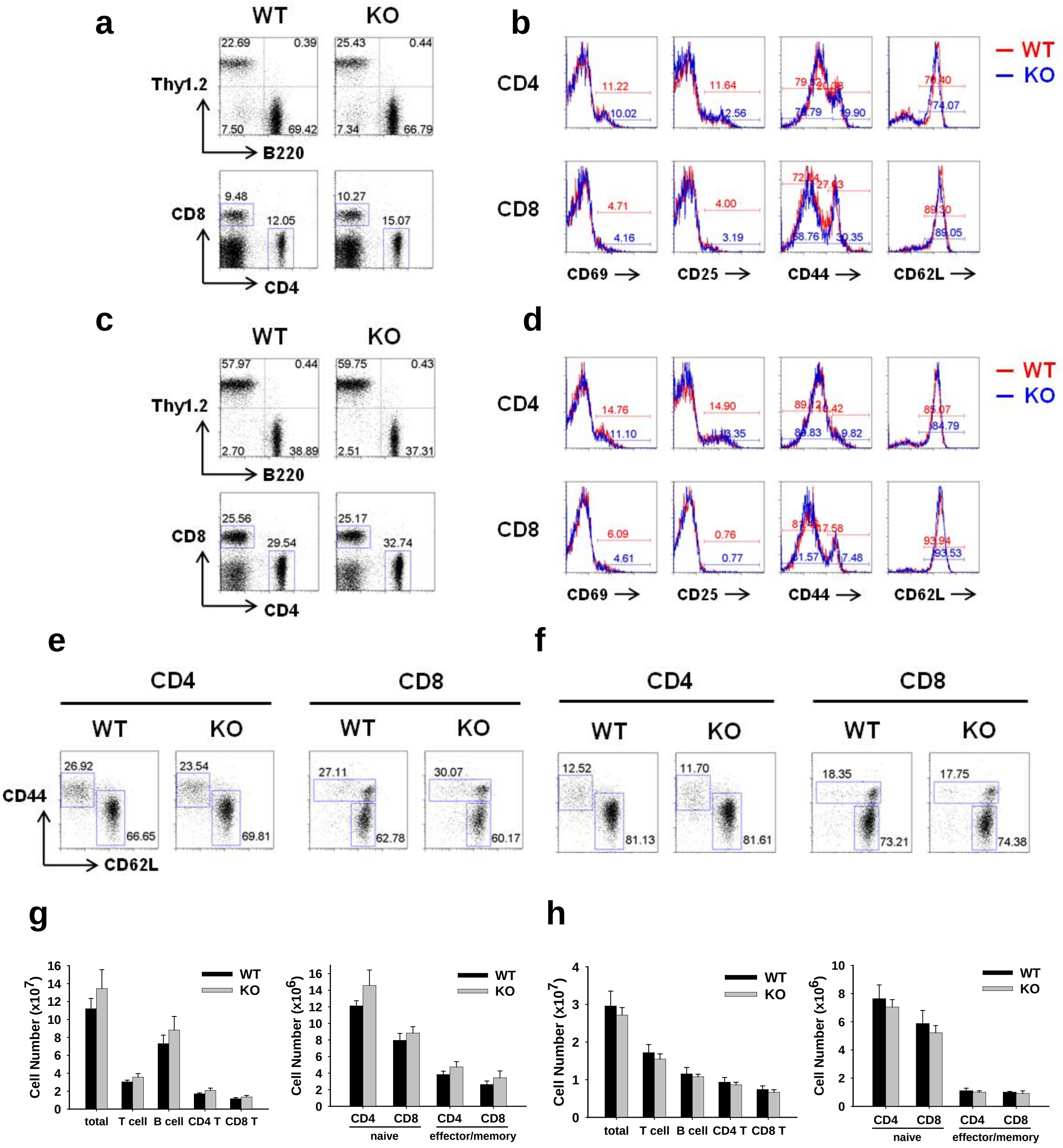

Supplementary Figure 10. Normal T cell population of spleen and lymph nodes in Ndrg1 knockout mice. lymphocyte profile by flow cytometry analysis of splenocytes (**a, b, e, and g**) or lymph node cells(**c, d, f, and h**). The percentages of cells of each subpopulation were displayed in the representative dot blots (**a, c, e, and f**). Activation markers(CD25, CD69) and differentiation markers(CD44, CD62L) were analysed for the gated $CD4^{+}$ or $CD8^{+}$ T cells and displayed as histograms (**b,d**) or dot blots(**e,f**). The absolute cell number of each subpopulation of 4 different mice was averaged (**g,h**). T cell, $Thy1.2^{+}$; B cell, $B220^{+}$; naïve, $CD44^{lo}$ $CD62L^{hi}$; effector/momory, $CD44^{hi}CD62L^{lo}$($CD4^{+}$) or $CD44^{hi}CD62L^{lo\text{-}hi}$($CD8^{+}$). The mice were 14-15 weeks old at the time of analysis. (error bars, s.e.m.)

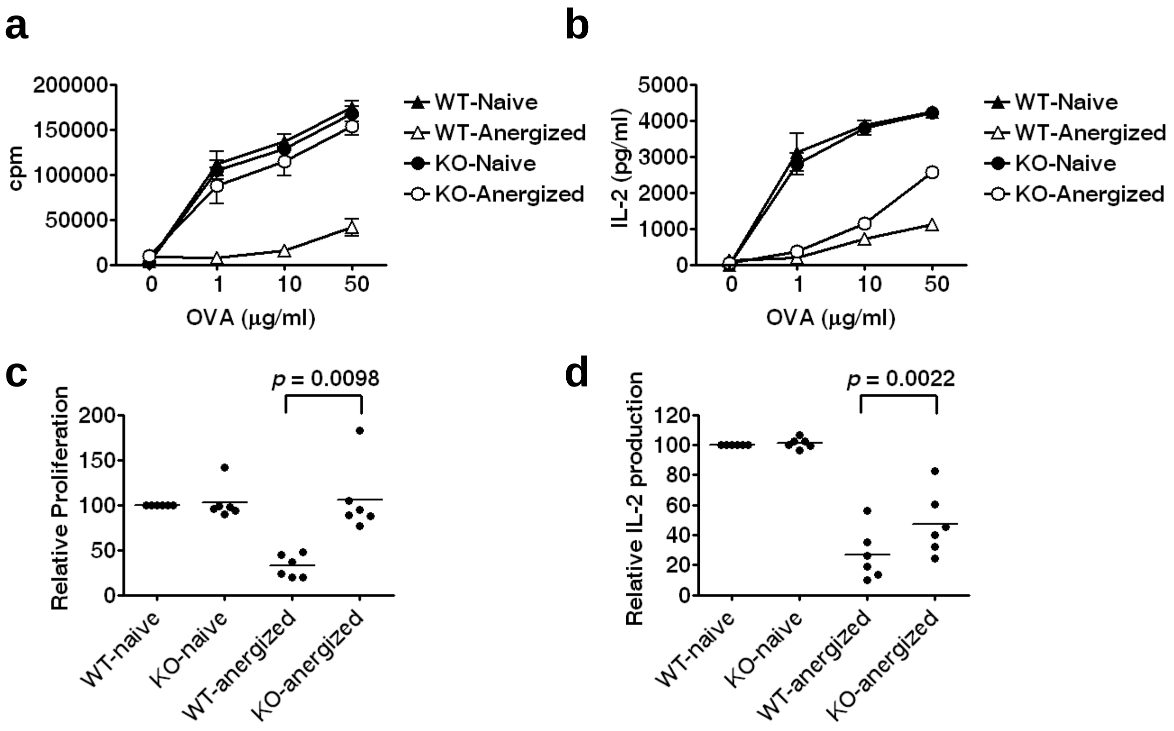

Supplementary Figure 11. Effect of Ndrg1 on peptide-induced *in vivo* anergy induction. (a-d) Proliferation and IL-2 production of in vivo anergized CD4+ T cells. The wild type OT-II or Ndrg1 knockout OT-II T cells were injected into congenic Thy1.1+ normal mice with subsequent injection of ovalbumin peptide. After 5d, OT-II T cells were isolated from the mice and stimulated with antigen plus APC. Proliferation (**a**) or IL-2 production (**b**) was compared with that of uninjected naïve wild type or Ndrg1 knockout OT-II T cells. Results from six independent experiments were plotted as relative proliferation (**c**) or relative IL-2 production (**d**). Relative proliferation = (cpm of the cells stimulated with 50µg ml^{-1} of OVA / cpm of WT naïve cells stimulated with 50µg ml^{-1} of OVA) x 100; Relative IL-2 production = (IL-2 produced from the cells stimulated with 50µg ml^{-1} of OVA / IL-2 produced from WT naïve cells stimulated with 50µg ml^{-1} of OVA) x 100. *P* values, Student's t-test. (error bars, s.d.)

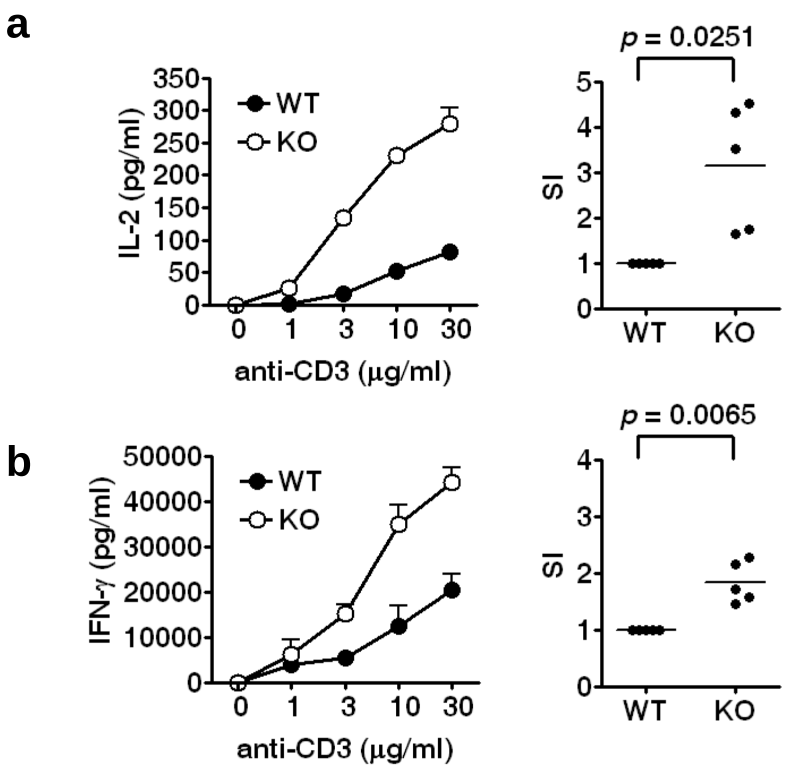

Supplementary Figure 12. Old Ndrg1-deficient CD8$^+$ T cells are hyperresponsive. IL-2 (**a**) and IFN-γ (**b**) production of CD8$^+$ T cells isolated from 1.5year-old wild type or Ndrg1-knockout mice upon stimulation with anti-CD3 plus anti-CD28. Left panel, dose-response curve of cytokine production from a representative set of experiments. Right panel, Degree of enhancement of cytokine production calculated as stimulation index (SI). SI = cytokine produced by Ndrg1-knockout T cells/cytokine produced by the wild type T cells stimulated with 10μg ml^{-1} (IL-2) or 30μg ml^{-1} (IFN-γ) of anti-CD3 plus anti-CD28. Data were from three independent experiments using 5 mice per group in total. (error bars, s.d.)

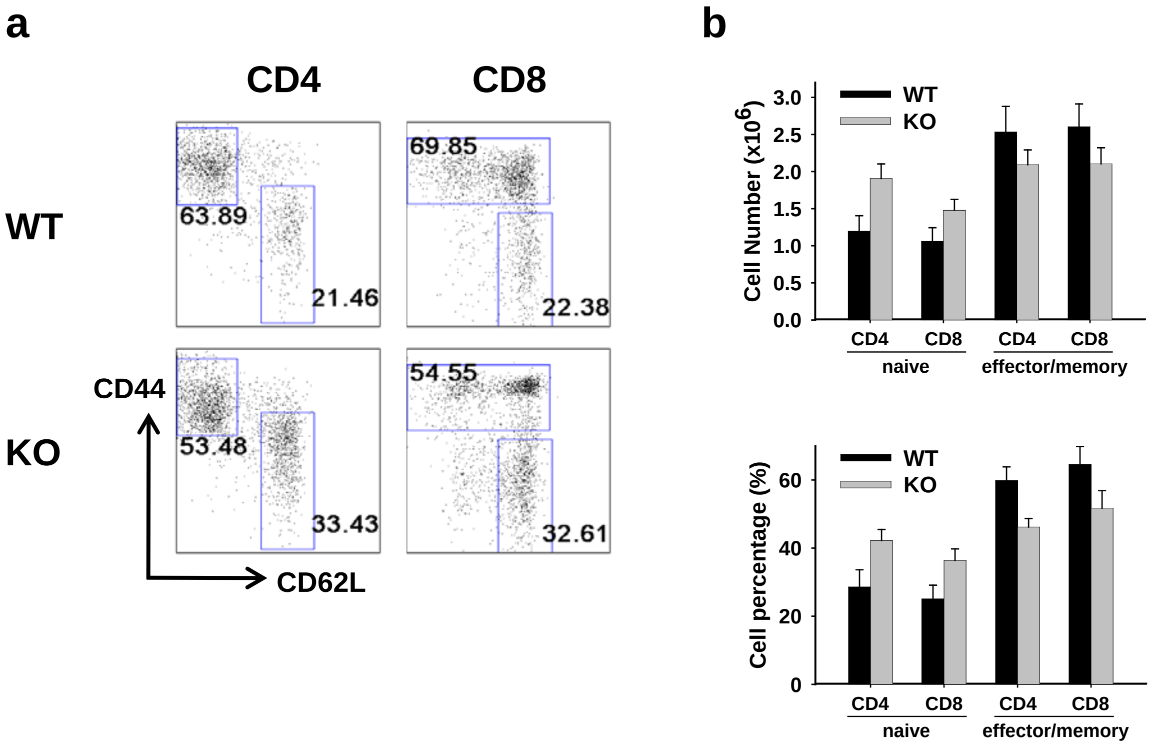

Supplementary Figure 13. T cell phenotype of old Ndrg1 knockout mice. Flow cytometry profile of CD44 and CD62L staining of $CD4^{+}$ or $CD8^{+}$ gated lymph node cells of approximately 1.5yr-old wild type or Ndrg1 knockout mice. The percentages of cells of each subpopulation were displayed in the representative dot blots (**a**). The absolute cell number or percentage of each subpopulation of 5 different mice was averaged (**b**). naïve, $CD44^{lo}$ $CD62L^{hi}$, effector/momory, $CD44^{hi}CD62L^{lo}$($CD4^{+}$) or $CD44^{hi}CD62L^{lo\text{-}hi}$($CD8^{+}$). (error bars, s.e.m.)

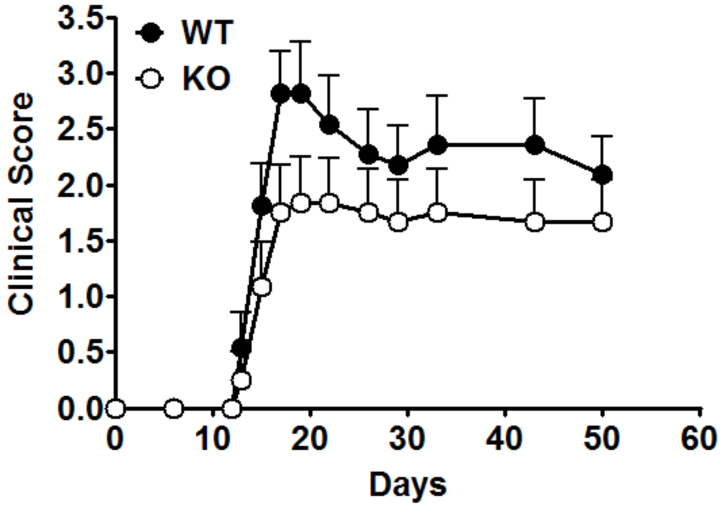

Supplementary Figure 14. EAE is not aggravated by Ndrg1-deficiency in young mice. EAE was induced in 12-14wk-old wild type or Ndrg1-knockout mice by subcutaneous injection of 200μg MOG peptides in CFA with 500μg Mycobacterium on day 1, supplemented by intravenous injections of 200ng pertussis toxin on days 1 and 3. The mice were observed regularly for clinical signs and the mean clinical score was measured. Pooled data from two independent experiments are shown. Five or six mice per group were used for each experiment. (error bars, s.e.m.)

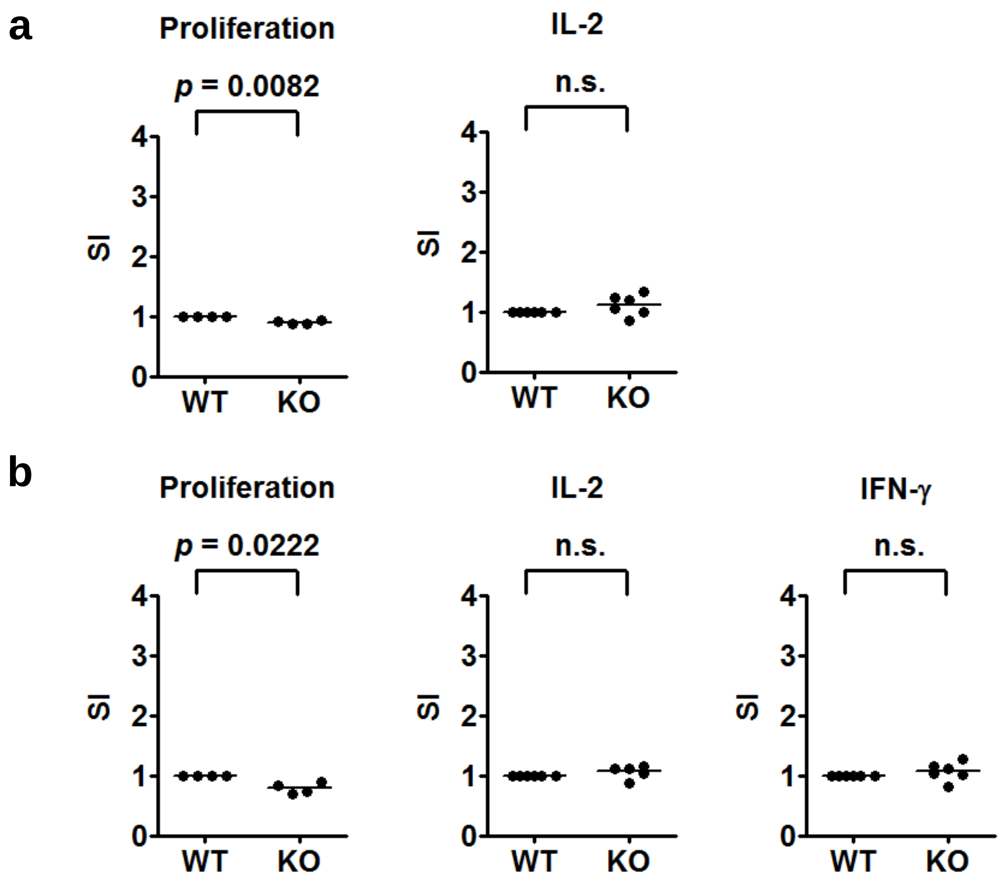

Supplementary Figure 15. Effector/memory T cell reactivity is not enhanced by Ndrg1-deficiency in young mice. (**a,b**) Proliferation and cytokine production of the naïve (**a**) or effector/memory CD4+ T cells (**b**) from 10-13 week-old wild type or Ndrg1-knockout mice upon stimulation with anti-CD3 (10µg ml^{-1}) plus anti-CD28 represented as stimulation index (SI). Data were from two to three independent experiments using 4-6 mice per group in total. n.s., not significant ($p > 0.05$); *P* values, Student's *t*-test.

Figure 1b

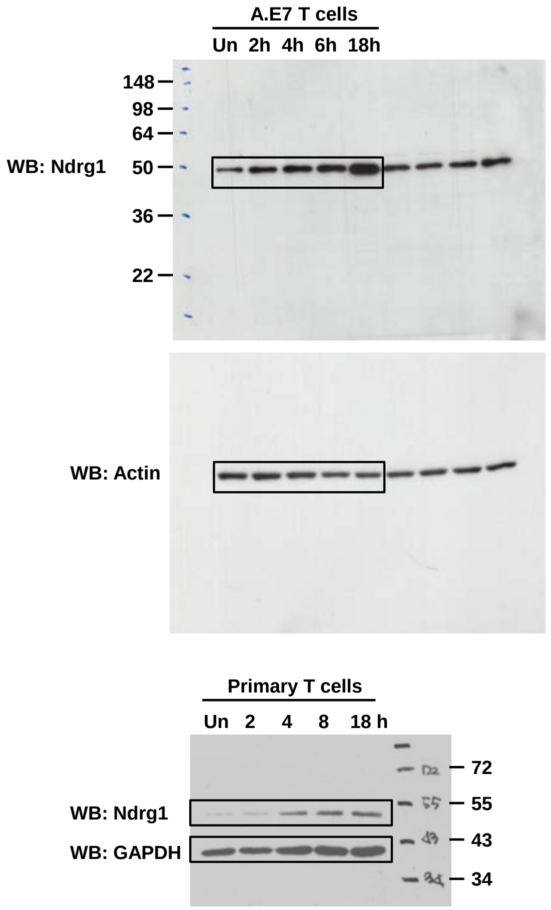

Figure 1c

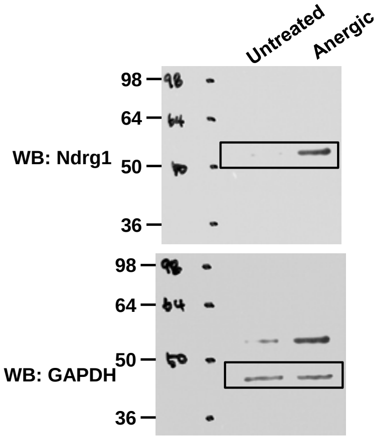

Figure 1d

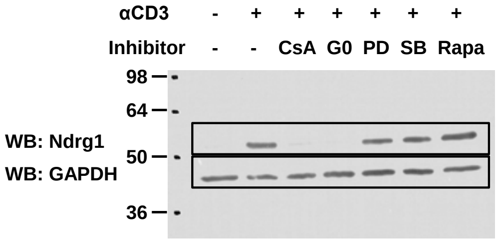

Figure 3d

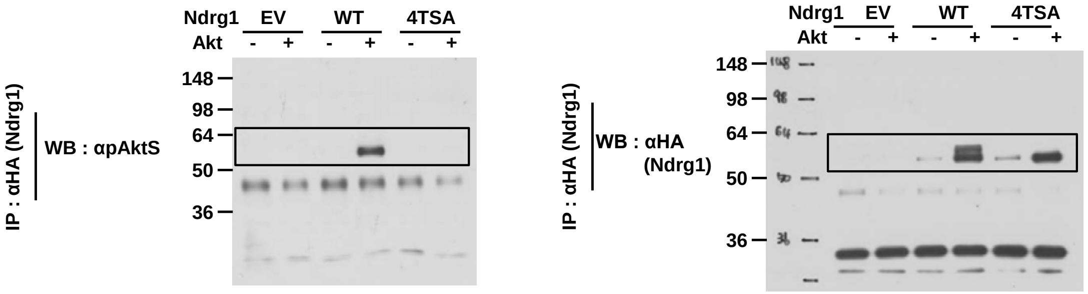

Supplementary Figure 16. Uncropped original scans.

Figure 4a

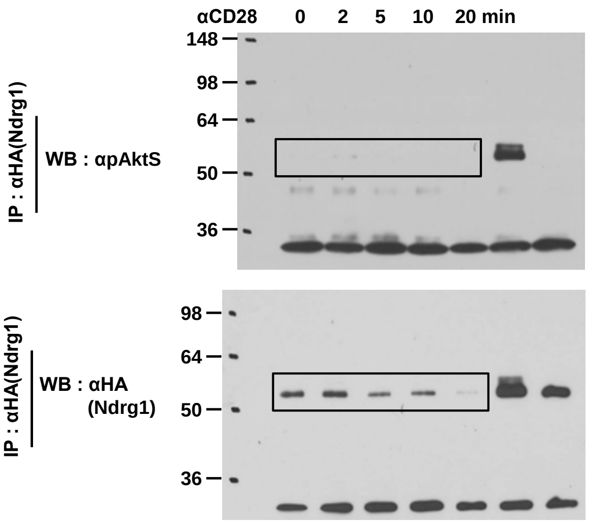

Figure 4b

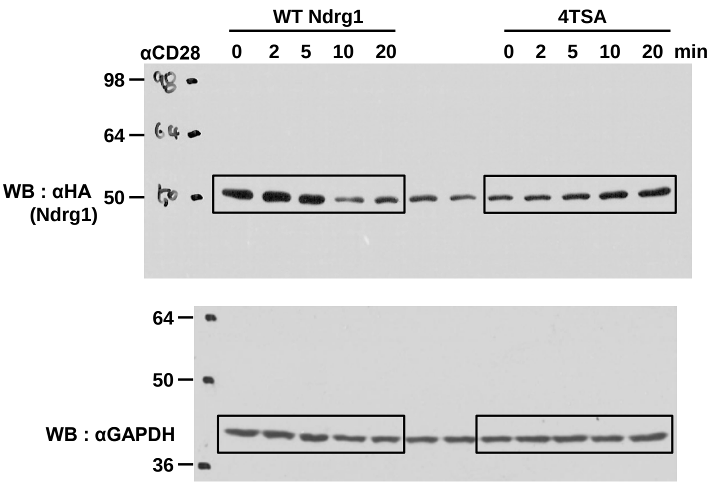

Figure 4c

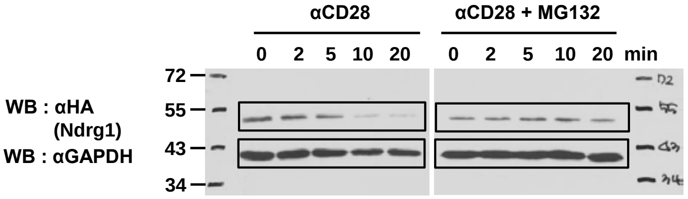

Figure 5a

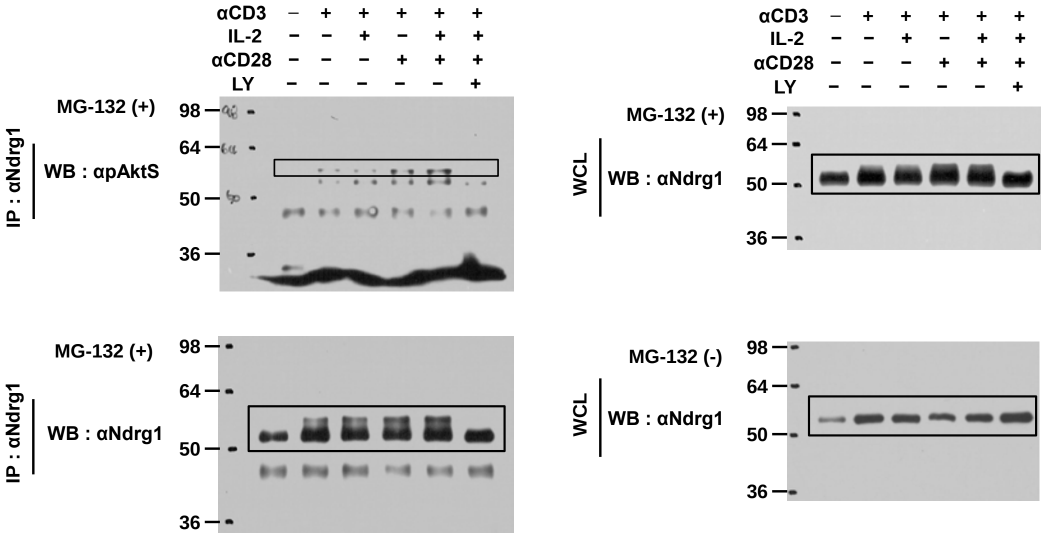

Supplementary Figure 16. Continued.

Figure 5b

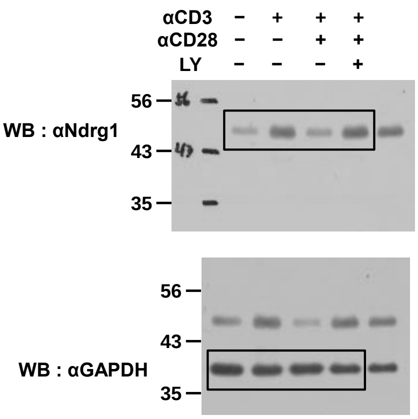

Figure 5d

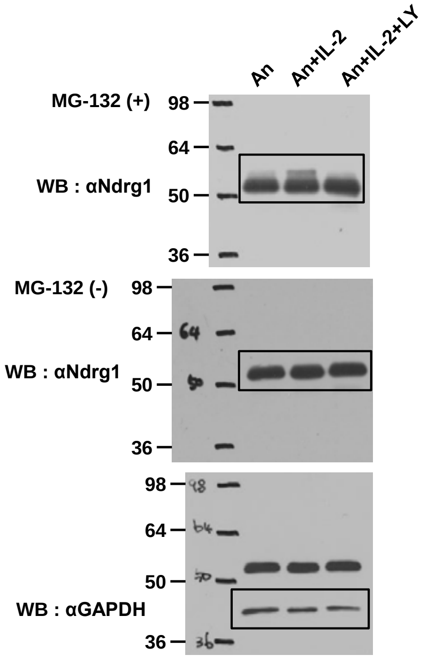

Figure 5e

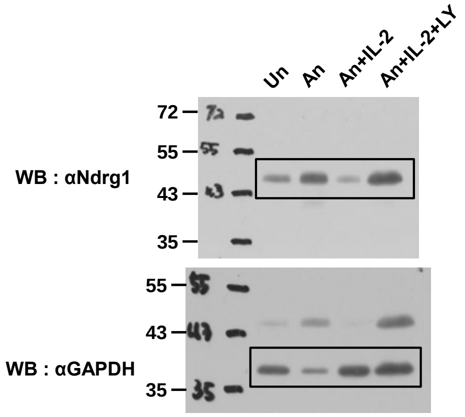

Figure 6d

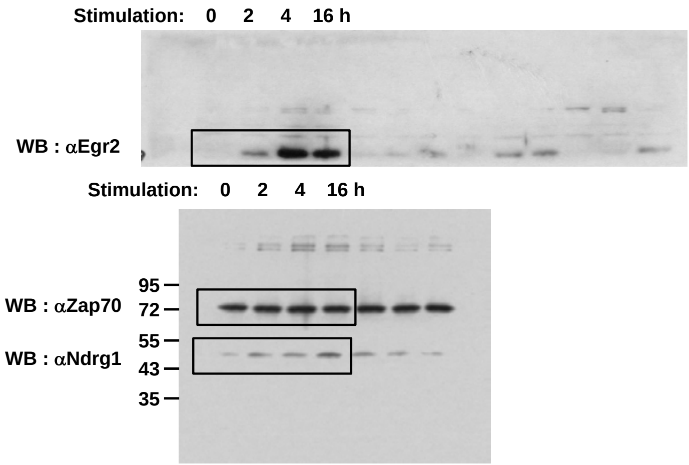

Supplementary Figure 3

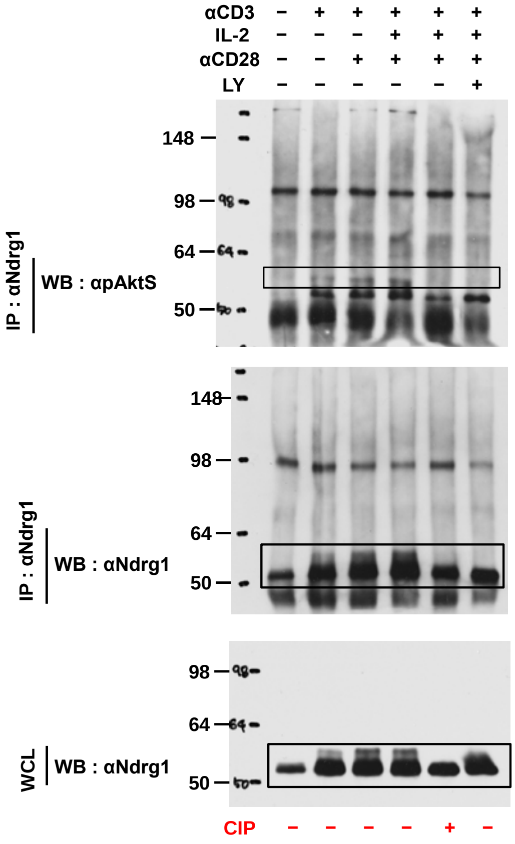

Figure 6e

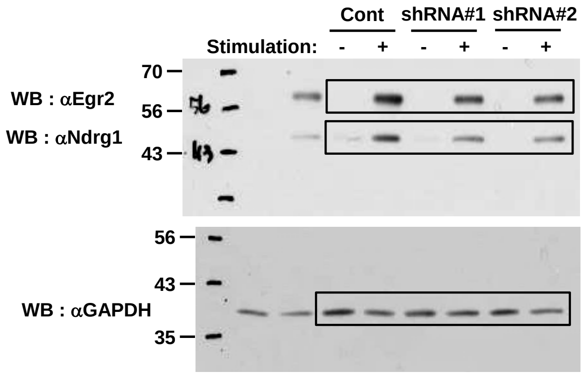

Supplementary Figure 16. Continued.

Supplementary Figure 5a

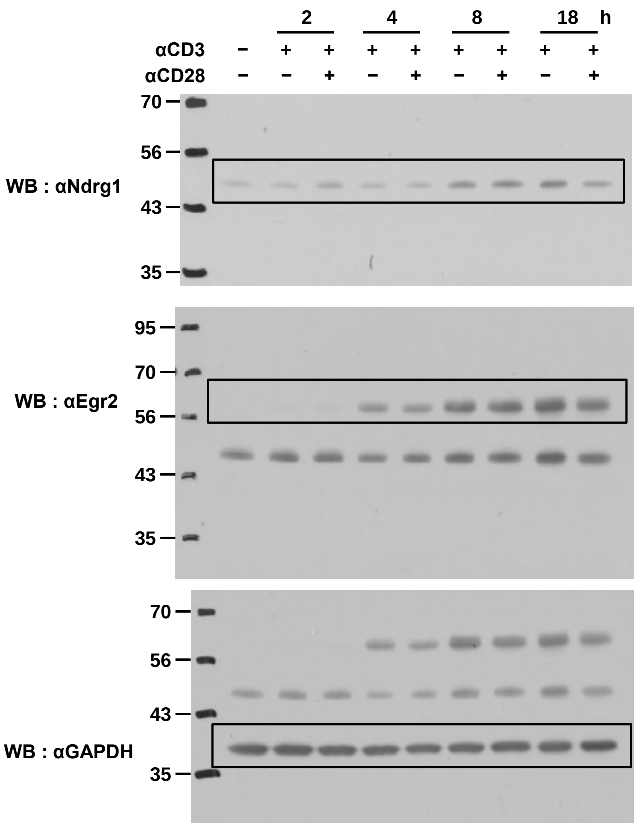

Supplementary Figure 5b

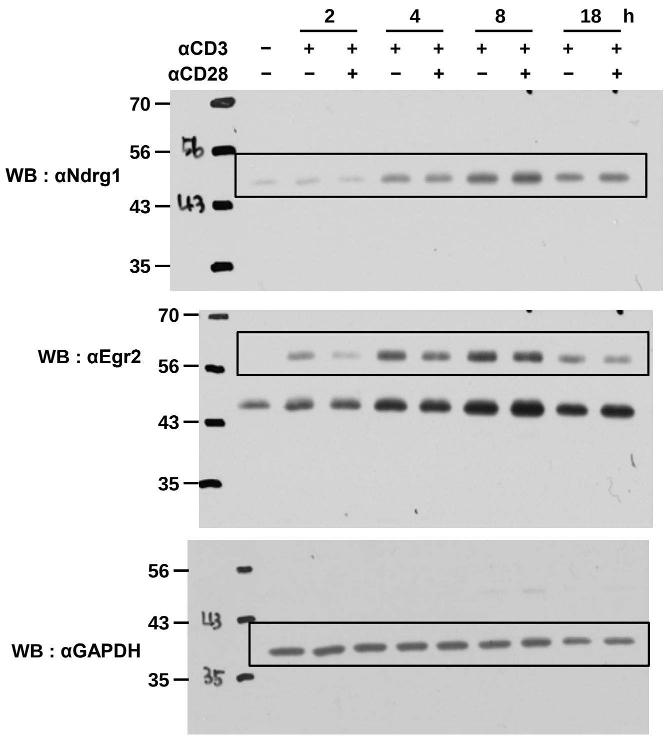

Supplementary Figure 6

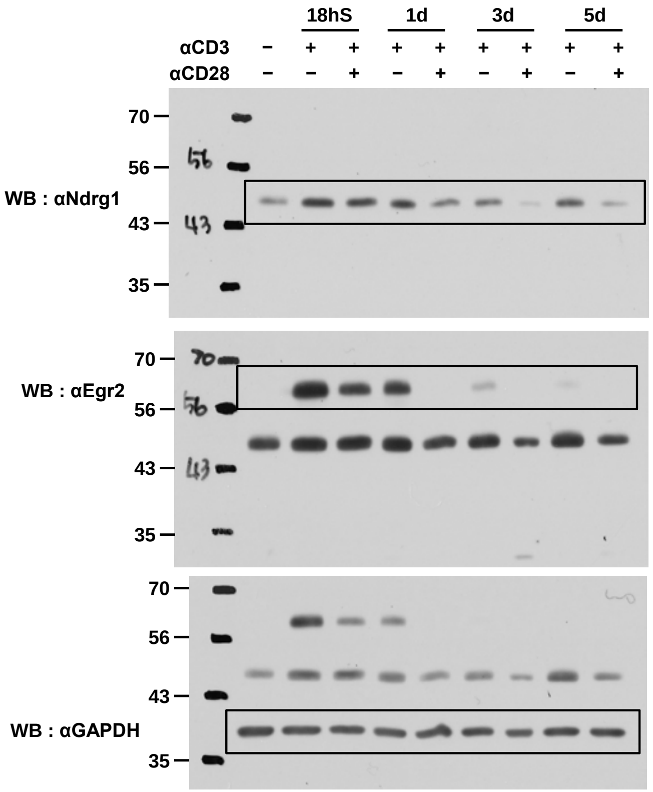

Supplementary Figure 7

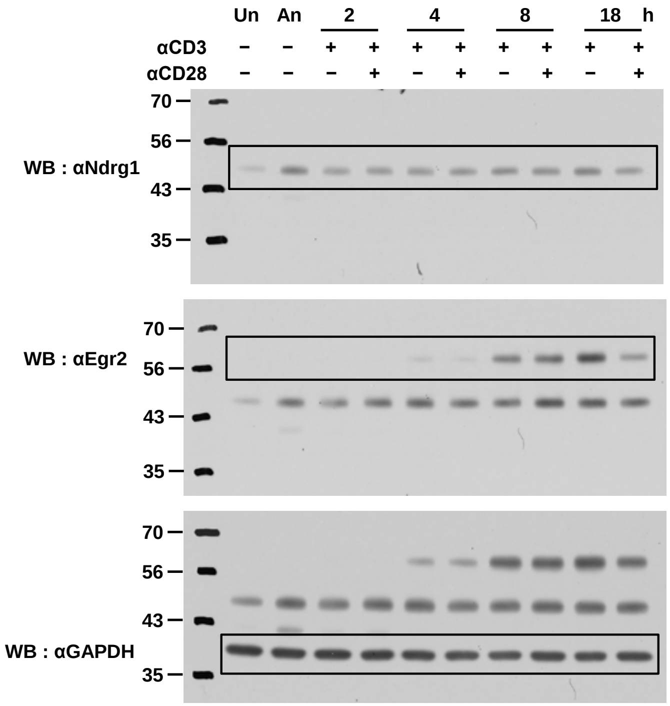

Supplementary Figure 16. Continued.

Supplementary Table 1. High-stringency selection of anergy-related genes

Affimetrix ID*	Gene name	Mock Avg Diff	Anergy Avg Diff	CsA Avg Diff	Gö9676 Avg Diff	comb Avg Diff	CsA/Aner <0.1	Go/Aner <0.1	Comb/Aner >0.4
102424_at	Mip3-alpha	51.2	9784.8	292.8	63.6	6813.9	0.036	0.005	0.693
99334_at	IFN-gamma	148.4	7075	813.2	85.6	3438.1	0.099	0.018	0.417
92248_at	Nurr1	42.4	2220.3	116.5	121.6	1090	0.058	0.060	0.495
92249_g_at	Nurr1	47.4	2537.1	147.1	136.1	1452	0.068	0.063	0.525
97106_at	Cot kinase (MKKK8)	-28	728.5	-26.9	-22.9	358.7	0.027	0.032	0.521
92415_at	41BBL	31.7	1099.7	-7.5	34.2	462.5	0.029	0.032	0.421
102661_at	EGR2	189.1	6058.6	643.9	156.9	2688.6	0.092	0.033	0.443
160464_s_at	Ndr1	19.5	581.9	20.2	23.5	544.3	0.036	0.043	1.032
102645_at	EST1	45.2	494.4	72.1	47.6	247.4	0.098	0.067	0.591
95506_at	Myosin Ic	15.8	550.2	119.7	67.1	302.9	0.098	0.098	0.500
163956_at	mSpry1	-92.7	5300.7	-45.6	-68.2	2057.1	0.022	0.0161	0.402
115499_g_at	EST2	50.2	1029.2	21.6	42.5	480.5	0.053	0.0535	0.471
115498_at	EST3	22.7	763.7	7.3	-19.5	297.2	0.069	0.0694	0.417
137028_at	EST4	197.9	2280.9	116.4	64.9	1395.3	0.085	0.085	0.581

Avg Diff, average difference : amount of mRNAs represented as fluorecent intensity.
Mock : mRNAs from unstimulated A.E7 cells
Anergy : pooled mRNAs from A.E7 cells stimulated with anti-TCR for 2, 4 and 6h.
CsA : pooled mRNAs from A.E7 cells stimulated with anti-TCR for 2, 4 and 6h in the presence of cyclosporin A.
Gö9676 : pooled mRNAs from A.E7 cells stimulated with anti-TCR for 2, 4 and 6h in the presence of Gö9676.
Comb : pooled mRNAs from A.E7 cells stimulated with anti-TCR for 2, 4 and 6h in the presence of PD98059, SB203580 and rapamycin.

CsA/Anergy = Avg Diff of CsA/Avg Diff of Anergy, **Go/Aner** = Avg Diff of Gö9676 /Avg Diff of Anergy, **Comb/Aner** = Avg Diff of Comb /Avg Diff of Anergy.

***Inclusion Criteria**: genes of which induction was downregulated more than 10 fold by CsA and Go treatment along with anti-TCR compared with anti-TCR treatment alone (CsA/Aner < 0.1 and Go/Aner < 0.1) and was downregulated less than 2.5 fold by Comb treatment along with anti-TCR compared with anti-TCR treatment alone (Comb/Aner > 0.4)
